# Dynamic regulation of drug biodistribution by turning tumors into decoys for biomimetic nanoplatform to enhance the chemotherapeutic efficacy of breast cancer with bone metastasis

**DOI:** 10.1002/EXP.20220124

**Published:** 2023-07-02

**Authors:** Cuixia Zheng, Dandan Zhang, Yueyue Kong, Mengya Niu, Hongjuan Zhao, Qingling Song, Qianhua Feng, Xingru Li, Lei Wang

**Affiliations:** ^1^ School of Pharmaceutical Sciences Zhengzhou University Zhengzhou P. R. China; ^2^ Henan Key Laboratory Targeting Therapy and Diagnosis for Critical Diseases Zhengzhou P. R. China; ^3^ Gynecology The Third Affiliated Hospital of Zhengzhou University Zhengzhou P. R. China; ^4^ Translational Medical Center of Huaihe Hospital Henan University Kaifeng P. R. China; ^5^ Henan International Joint Laboratory of Ovarian Malignant Tumor Zhengzhou P. R. China

**Keywords:** analgesia, biomimetic nanoplatform, breast cancer with bone metastasis, pyroptosis

## Abstract

Breast cancer with bone metastasis accounts for serious cancer‐associated pain which significantly reduces the quality of life of affected patients and promotes cancer progression. However, effective treatment using nanomedicine remains a formidable challenge owing to poor drug delivery efficiency to multiple cancer lesions and inappropriate management of cancer‐associated pain. In this study, using engineered macrophage membrane (EMM) and drugs loaded nanoparticle, we constructed a biomimetic nanoplatform (EMM@DJHAD) for the concurrent therapy of bone metastatic breast cancer and associated pain. Tumor tropism inherited from EMM provided the targeting ability for both primary and metastatic lesions. Subsequently, the synergistic combination of decitabine and JTC801 boosted the lytic and inflammatory responses accompanied by a tumoricidal effect, which transformed the tumor into an ideal decoy for EMM, resulting in prolonged troop migration toward tumors. EMM@DJHAD exerted significant effects on tumor suppression and a pronounced analgesic effect by inhibiting µ‐opioid receptors in bone metastasis mouse models. Moreover, the nanoplatform significantly reduced the severe toxicity induced by chemotherapy agents. Overall, this biomimetic nanoplatform with good biocompatibility may be used for the effective treatment of breast cancer with bone metastasis.

## Graphical abstract

The schematic diagram of EMM@DJHAD NPs destroys tumor cells via the synergism of DAC and JTC. The former enriches the level of gasdermin E in tumor by demethylation of the DFNA5 gene and the latter activates the caspase‐3 pathway, triggering pyroptosis of tumor cells. Importantly, JTC‐mediated µ‐opioid receptor (MOR) inhibition is responsible for both tumor suppression and pain relief. Interestingly, highly‐inflammatory pyroptosis makes tumor an ideal decoy for EMM, amplifying the nanodrug targeting effect.

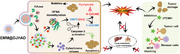

## 1 INTRODUCTION

Metastasis is the most devastating stage of cancer progression that is responsible for the high mortality rate of breast cancer.^[^ ^1^ ^]^ Bone organ is a particularly common site affected by metastatic breast cancer.^[^ ^2^ ^]^ Bone metastasis seriously impairs the patient's life quality by inducing severe pain and other skeletal‐related events.^[^ ^3^ ^]^ Cancer‐associated pain is not just the outcome of cancer, but also a promoter of cancer progression, indicating that concurrent therapy of cancer and associated pain may aid in improving the efficacy of comprehensive cancer treatment.^[^ ^4^ ^]^ Therefore, there is an urgent need to develop efficient therapeutic strategies for the treatment of breast cancer with bone metastases.

Local surgery and radiotherapy are the two major pillars of primary breast cancer management, but they cannot completely eradicate bone metastases with multiple metastatic lesions.^[^ ^1^, ^5^ ^]^ Systemic chemotherapy, combined with analgesic and anti‐bone absorption adjuvant therapy, is a cornerstone for treatment of metastatic breast cancer.^[^ ^6^ ^]^ Opioids are commonly used analgesics to manage cancer‐associated pain by acting on various opioid receptors clinically.^[^ ^7^ ^]^ However, high expression of opioid receptors was observed in breast cancer tissue.^[^ ^4^, ^8^ ^]^ Opioid receptors agonists can promote tumor progression and metastasis by further upregulating opioid receptors expression in tumor tissue. On the contrary, opioid receptors antagonists exhibit a potent inhibition of tumor gross growth and cancer associated pain^[^ ^9^ ^]^ Therefore, µ‐opioid receptor (MOR) antagonists are a better choice for their positive effects on tumor suppression and pain relief.^[^ ^10^ ^]^

Non‐specific distribution of therapeutic drugs may cause undesirable side effects.^[^ ^11^ ^]^ Various nanomaterials have been developed as drug delivery carriers to improve the tumor‐targeting abilities of drugs and protect the normal tissues from undesired damage.^[^ ^12^ ^]^ Nevertheless, it is difficult to simultaneously target multiple cancer lesions because of the distinct traits of primary and bone metastatic tumors.^[^ ^6^, ^13^ ^]^ For instance, majority of nano‐drug‐delivery systems are effective in well‐vascularized tumors.^[^ ^14^ ^]^ In bone metastases with poorly‐built vasculature, especially multiple micro‐metastatic nodules, inadequate drug concentration makes it difficult to achieve satisfactory therapeutic effects.^[^ ^15^ ^]^ Similarly, bone‐seeking moiety modification can overcome poor blood supply in delivering therapeutic agents to bone metastatic regions, but it is difficult to use these systems for the simultaneous treatment of primary and bone metastatic tumors.^[^ ^16^ ^]^

Cell membrane biomimetic drug delivery vehicles, especially macrophage membrane (MM)‐based drug carriers, may be alternatives to circumvent the above‐mentioned limitations because MM can drive the drug vectors to accumulate in inflammatory locations.^[^ ^17^ ^]^ Inflammation is one of the hallmarks and a “double‐edged sword” in cancer.^[^ ^18^ ^]^ Chronic inflammation is precursor to most cancers and strongly influences cancer development by promoting malignant progression and metastasis.^[^ ^19^ ^]^ Therefore, controlling inflammation is an important approach for efficient anti‐cancer treatment.^[^ ^20^ ^]^ For instance, pyroptosis, a type of programmed cell necrosis, is a form of cell death triggered by tumor‐inhibiting inflammation.^[^ ^21^ ^]^

**SCHEME 1:**
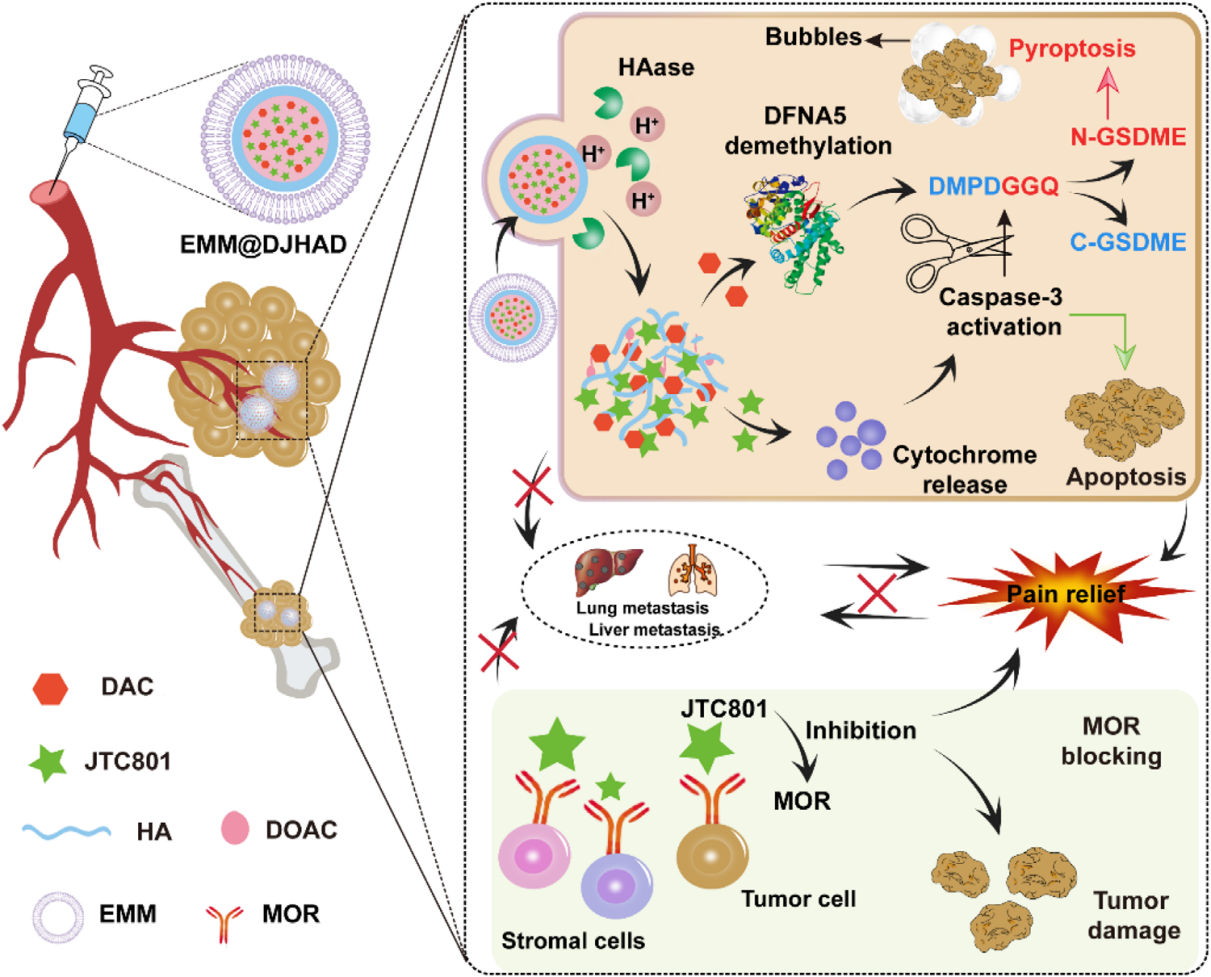
Illustration of the antitumor effects of EMM@DJHAD. EMM, engineered macrophage membrane.

In this study, we constructed engineered M1 macrophage membrane (EMM) biomimetic nanoplatform (EMM@DJHAD) to deliver JTC801 and methyltransferase inhibitor decitabine (DAC) for the treatment of breast cancer with bone metastasis. As shown in Scheme 1, EMM@DJHAD showed active targeting ability in breast cancer with bone metastasis. At the tumor site, DAC increases the level of gasdermin E (GSDME) via DFNA5 gene demethylation. JTC801 activated caspase‐3 to cleave GSDME, triggering tumor pyroptosis. Interestingly, highly‐inflammatory pyroptosis made the tumor an ideal decoy for EMM, achieving amplified tumor‐targeting efficiency. Importantly, the nanoplatform exhibited outstanding inhibitory effects on inhibition of tumor growth, metastasis, and analgesic activity in breast cancer and bone metastasis mouse models. Therefore, this biomimetic nanoplatform with good biocompatibility may be used for the effective treatment of breast cancer with bone metastasis.

## 2 RESULTS AND DISCUSSION

### 2.1 Preparation and characterization of EMM@DJHAD

The amphiphilic hyaluronic acid (HA)‐deoxycholic acid (DOCA) conjugate was synthesized by grafting aminated deoxycholic acid (DOCA‐NH_2_) onto an HA backbone, and the synthesis procedure was shown in Figure S1. The increase in the methylene absorption peak at 2919 cm^−1^ in Fourier transform infrared spectroscopy (FTIR) (Figure S2) and characteristic peaks for DOCA at 0.67–1.60 ppm in proton nuclear magnetic resonance (^1^H NMR) spectroscopy (Figure S3) confirmed the successful synthesis of the HA‐DOCA conjugate.^[^ ^22^ ^]^

**FIGURE 1:**
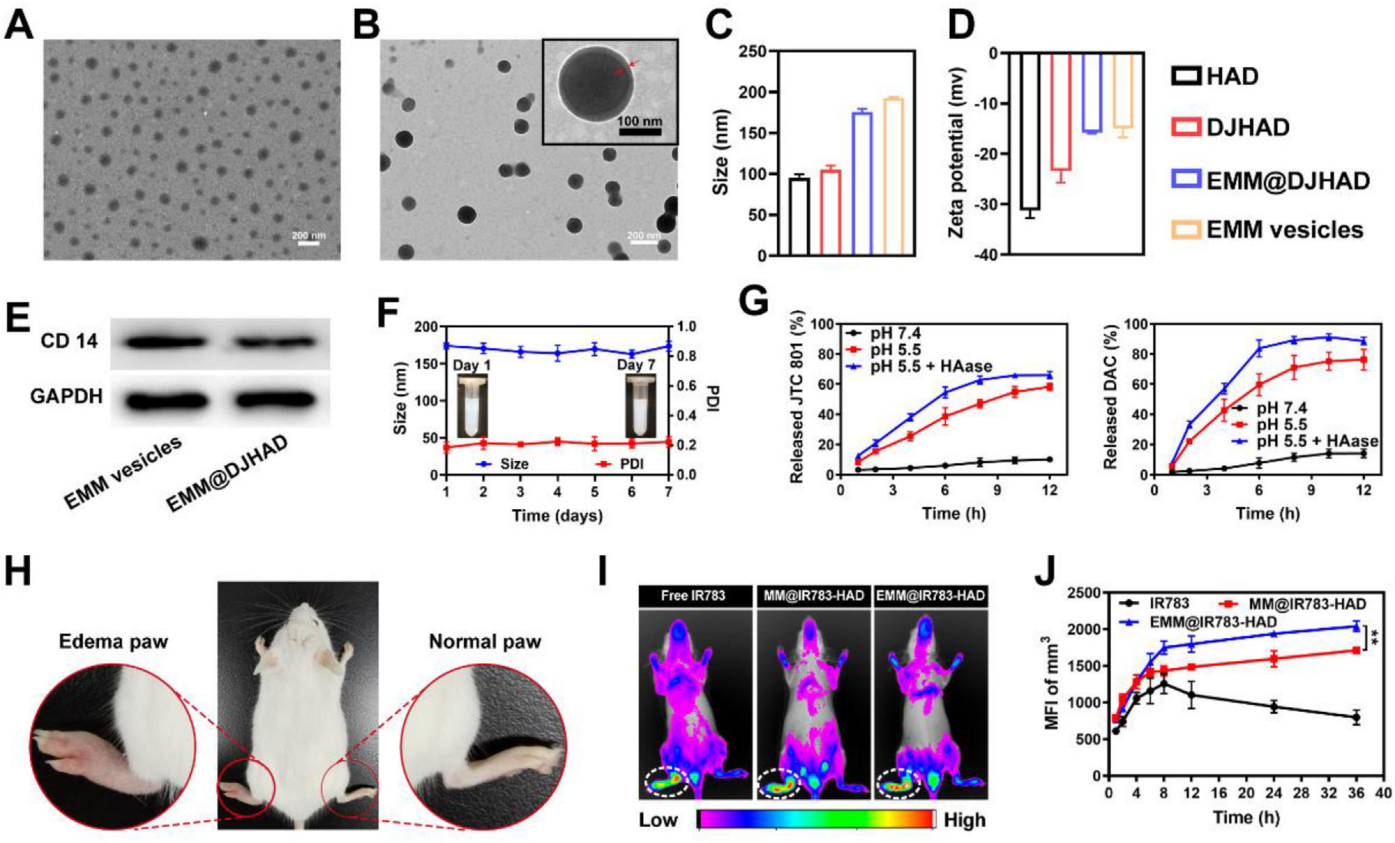
Preparation and characterization of EMM@DJHAD. (A) Transmission electron microscope (TEM) image of DJHAD. (B) TEM image of EMM@DJHAD. (C) Size distribution. (D) Zeta potential. (E) CD14 on EMM and EMM@DJHAD. (F) Size and polydispersity index (PDI) of EMM@DJHAD NPs, (*n* = 3). (G) Release profile of JTC801 and DAC (*n =* 3). Insets are the photographs of EMM@DJHAD. (H) Paw edema model in Balb/c mice. Left, edema paw. Right, normal paw. (I) In vivo near‐infrared (NIR) images at 24 h. (J) Fluorescence intensity of edema paw at different time points. EMM, engineered macrophage membrane.

In an aqueous solution, the HA‐DOCA conjugate was self‐assembled into HAD nanoparticles (NPs). Transmission electron microscope (TEM) analysis revealed that HAD NPs exhibited spherical morphology (Figure S4A) with the mean size of ∼ 100 nm (Figure S4B). Drugs loaded NPs (DJHAD) were obtained using the same self‐assembly process. The characteristic absorbance peaks (Figure S5) of DAC and JTC801 in the ultraviolet−visible light−near‐infrared (UV–vis‐NIR) absorption spectrum confirmed that the two drugs were introduced into DJHAD. The encapsulation efficiencies of DJHAD were 42.1 ± 1.9% for JTC801 and 37.8 ± 2.6% for DAC. The drug loading efficiencies of JTC801 and DAC were 11.5 ± 2.3% and 7.7 ± 1.5%, respectively. Figure 1A and Figure S4C displayed the morphology and mean size of DJHAD, which showed no significant difference from HAD NPs.

M1 macrophages were the donors of EMM. High levels of CD86, tumor necrosis factor‐α (TNF‐α), and interleukin 12 (IL‐12) (Figure S6) confirmed that RAW267.4 macrophages were polarized to M1 macrophages by lipopolysaccharide (LPS) and interferon γ (IFN‐γ).^[^ ^23^ ^]^ Subsequently, EMM was extracted and coated onto the surface of DJHAD to form EMM@DJHAD. As displayed in Figure 1B, EMM@DJHAD NPs exhibited a spherical core–shell structure with an increased particle size compared to DJHAD (Figure 1C and Figure S7). The zeta potential of EMM@DJHAD was less negative than that of DJHAD, but similar to that of EMM (Figure 1D). Furthermore, western blotting analysis confirmed the existence of CD14, an endotoxin receptor on macrophages, on the surface of both EMM and EMM@DJHAD (Figure 1E). Sodium dodecyl sulfate polyacrylamide gel electrophoresis demonstrated that the presence of key surface proteins on EMM@DJHAD matched well with that of EMM (Figure S8), which further confirmed the successful synthesis of EMM@DJHAD.

EMM@DJHAD exhibited good storage stability with no significant changes in the appearance or particle size over 7 days (Figure 1F). Additionally, negligible hemoglobin release and a low hemolysis rate (< 5%) were observed in the hemolysis assay (Figure S9), indicating the good blood compatibility of EMM@DJHAD. To investigate the drug release profile, different buffer solutions were used to simulate blood circulation (pH 7.4) and the tumor microenvironment (pH 5.5, 0.5 mg mL^‐1^ HAase). Both JTC801 and DAC were released from EMM@DJHAD in a pH‐ and HAase‐dependent manner, with < 20% released at pH 7.4, within 12 h (Figure 1G). The cumulative release percentage of JTC801 was 66.3 ± 3.2% over 12 h and that of DAC was 89.1 ± 2.9%.

In order to verify inflammatory tendency of the biomimetic nanoplatform, we assessed its biodistribution in an inflammatory model. First, a murine paw edema model (left hind paw) was established via an intraplantar injection of the complete freund's adjuvant (CFA).^[^ ^24^ ^]^ Redness and swelling were observed at the inflamed sites (Figure 1H). IR783, MM@IR783‐HAD, and EMM@IR783‐HAD were injected via the tail vein. Fluorescence was monitored non‐invasively for up to 36 h via NIR fluorescence imaging. As displayed in Figure S10A and Figure 1I, compared to normal paws, increased fluorescence signals were observed in edema paws post‐injection of IR783, MM@IR783‐HAD, and EMM@IR783‐HAD. Interestingly, EMM@IR783‐HAD exhibited a superior IR783 signal compared to MM@IR783‐HAD and free IR783 in the inflamed paw after 8 h (Figure 1J). Moreover, the ex vivo fluorescence signal of the inflamed paws displayed a similar distribution tendency (Figure S10B,C). A stronger fluorescence signal was observed in liver after injection of free IR783. Compared with MM camouflaged NPs, EMM@IR783‐HAD showed a stronger accumulation ability in edema paws, which might be attributed to the recruitment of inflammation on EMM.

### 2.2 In vitro therapeutic efficacy of EMM@DJHAD

**FIGURE 2:**
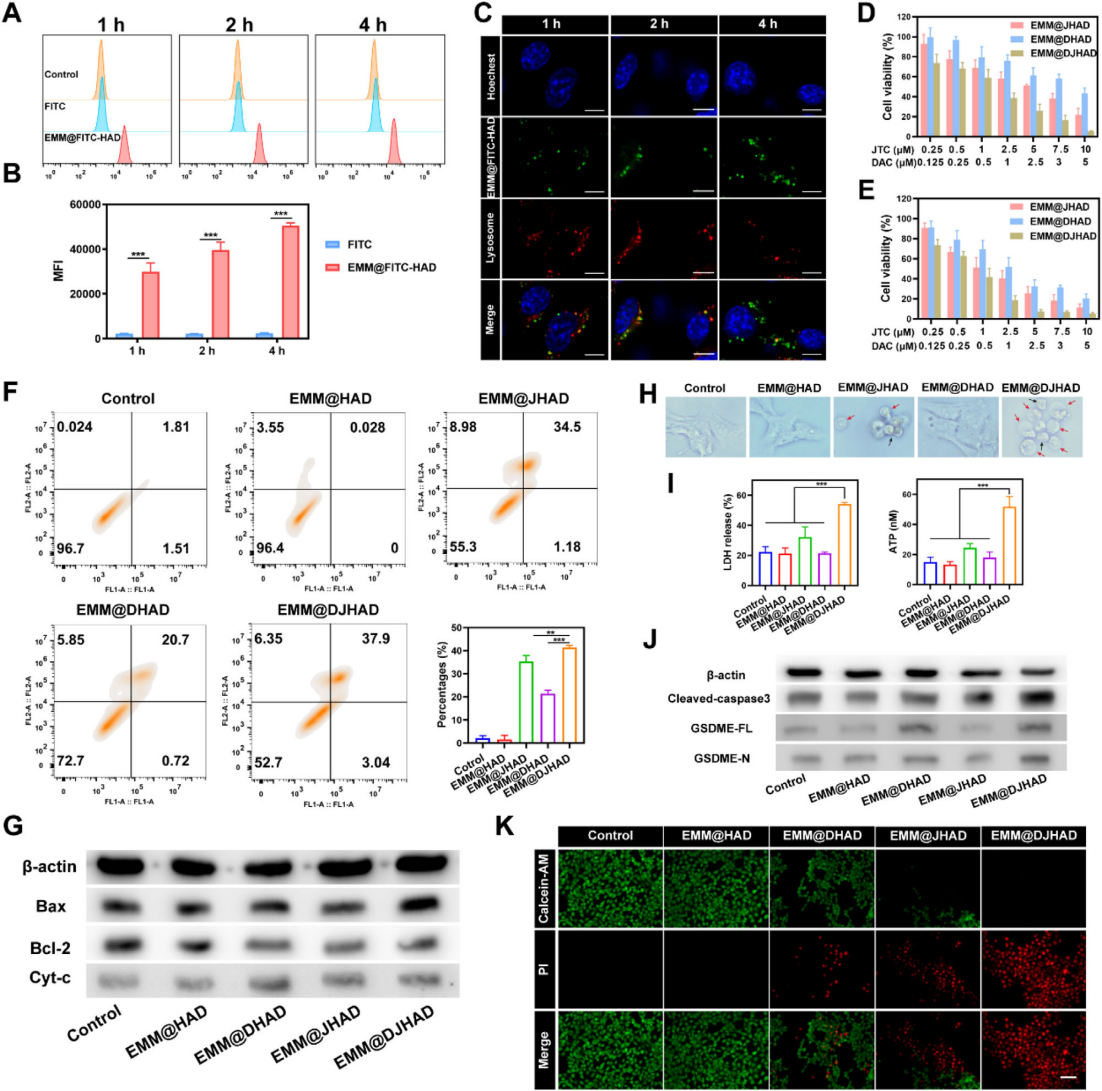
In vitro pyroptosis and antitumor performance of EMM@DJHAD. (A) Cellular uptake of fluorescein isothiocyanate (FITC) and EMM@FITC‐HAD. (B) Fluorescence intensity of FITC. (C) Lysosomal escape experiment, scale bar: 7.5 µm. Viability of 4T1 cells at (D) 24 and (E) 48 h post‐treatment. Values are represented as mean ± standard deviation (SD) (*n = 3*). (F) Flow cytometry apoptosis analysis and the percentages of early apoptotic plus late apoptotic cells. (G) Expression levels of apoptosis‐related proteins. (H) Representative bright‐field microscopy images of 4T1 cells. Red arrows, pyroptotic cells. Black arrows, apoptotic cells. (I) Released lactate dehydrogenase (LDH) and adenosine triphosphate (ATP) in cell culture supernatant (*n = 3*). (J) Expression levels of cleaved caspase‐3, GSDME‐FL, and GSDME‐N. (K) Fluorescence images of 4T1 cells stained with calcein AM/PI, scale bar, 100 µm. Data were presented as the mean ± SD. Statistical analysis was conducted using one‐way analysis of variance (ANOVA). *p < 0.05, **p < 0.01, and ***p < 0.001. EMM, engineered macrophage membrane.

The cellular uptake efficiency and endocytic pathways of the EMM biomimetic nanoplatform were assessed using flow cytometry and confocal laser scanning microscope (CLSM), respectively. Flow cytometry analysis demonstrated that fluorescein isothiocyanate (FITC) labeled NPs (EMM@FITC‐HAD) reached a high level within 1 h and showed time‐dependent cellular uptake (Figure 2A,B). As indicated in Figure 2C, lysotracker fluorescent signals merged with EMM@FITC‐HAD after incubation for 2 h, suggesting that EMM@FITC‐HAD NPs were endocytosed via the lysosome pathway. Four hours later, the expanded distribution of EMM@FITC‐HAD signals was observed in cytoplasm, but decreased in lysosomes. The above phenomenon demonstrated that EMM@HAD not only accelerated the cellular uptake efficiency, but also promoted lysosome escape of the nanoplatform.

Next, we investigated the in vitro cytotoxicity of EMM@HAD, EMM@JHAD, EMM@DHAD, and EMM@DJHAD toward 4T1 cells. As shown in Figure 2D,E, both EMM@DHAD and EMM@JHAD exhibited dose‐ and time‐dependent cytotoxicity. A marked synergistic effect of DAC and JTC801 was observed in EMM@DJHAD group, and the population of live cells was less than 20% after incubation for 24 h. Moreover, we detected cell apoptosis based on annexin V‐FITC/propidium iodide (PI) detection. In Figure 2F, approximately 41% of the cancer cells were apoptotic after treatment with EMM@DJHAD, which was significantly higher than that in cells treated with EMM@DHAD (∼ 21%) and EMM@JHAD (∼ 35%). In line with the above results, EMM@DJHAD induced a higher expression of apoptosis‐related proteins, such as cytoplasm c (Cyt‐c), Bcl‐2, Bax, and cleaved caspase‐3, as shown in Figure 2G–J and Figure S11.

To explore the synergistic mechanism of DAC and JTC801, EMM@DJHAD induced pyroptotic effect was investigated in a series of experiments. First, the microscopic features of pyroptosis were observed using an optical microscope. As shown in Figure 2H, large bubbles from the 4T1 cells membrane were observed to verify pyroptosis. Subsequently, the leakage of intracellular content was verified by monitoring the lactate dehydrogenase (LDH) and adenosine triphosphate (ATP) levels in cell supernatant. Results in Figure 2I showed that the combination of DAC and JTC801 significantly increased the release of pyroptotic indicators. Next, pyroptosis‐associated proteins were detected and the results were displayed in Figure 2J and Figure S12. Full‐length GSDME (GSDME‐FL) expression levels were increased in the EMM@DHAD and EMM@DJHAD groups, but GSDME‐N terminal (GSDME‐N) expression levels were increased only in EMM@DJHAD group. Additionally, high cell coverage revealed that EMM@DJHAD significantly increased pyroptotic cell percentage (Figure S13). To further examine EMM@DJHAD‐induced cytotoxicity, 4T1 cells were stained with calcein‐AM (green) and PI (red) to detect the live and dead cells. After treatment with EMM@DJHAD, a large amount of 4T1 cells died, as indicated by the strong red fluorescence, while negligible red staining was observed in the control group. The above observations concurred with the obvious cytotoxicity of EMM@DJHAD.

### 2.3 Tumor inhibition efficacy in transplanted breast cancer

**FIGURE 3:**
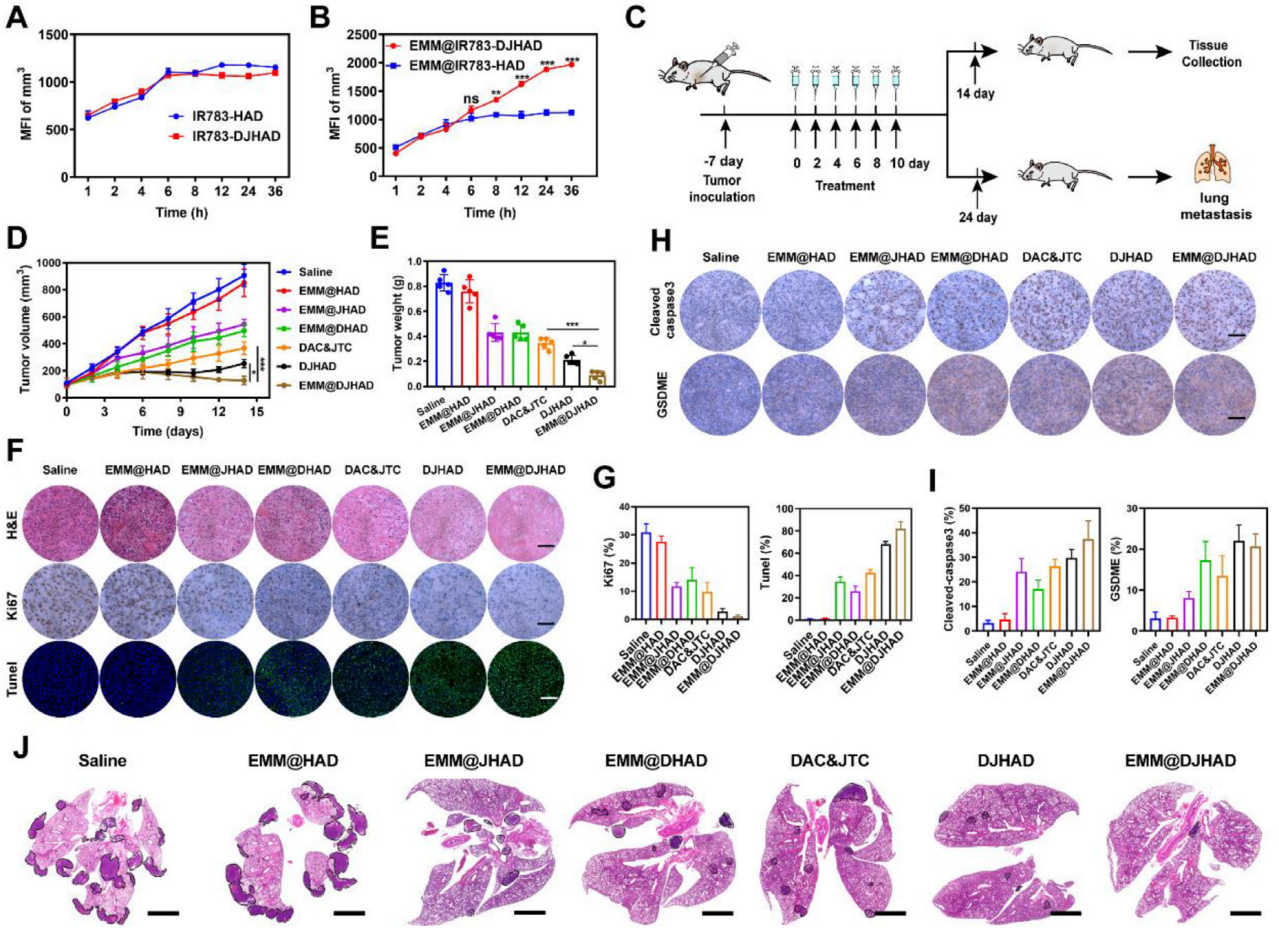
Antitumor efficacy in transplanted breast cancer. (A,B) MFI at tumor site. (C) Timeline of treatment schedule. (D) Tumor growth curves (*n =* 5). (E) Tumor weight (*n =* 5). (F) Hematoxylin and eosin (H&E) staining, Ki67, and transferase dUTP nick‐end labeling (TUNEL) staining. Scale bar: 200 µm. (G) Quantification of Ki67 and TUNEL staining (*n =* 3). (H) Expression levels of cleaved caspase‐3 and GSDME in tumor tissues. Scale bar: 200 µm. (I) Quantification of cleaved caspase‐3 and GSDME positive cells (*n =* 3). (J) H&E staining micrographs of lungs. Scale bar, 2000 µm. Data were presented as mean ± SD. Statistical analysis was conducted using one or two‐way analysis of variance (ANOVA). *p < 0.05, **p < 0.01, and ***p < 0.001.

First, the biodistribution of the nanoplatform was investigated in a 4T1 tumor‐bearing mouse model. Identical doses of IR783‐labeled HAD, DJHAD, EMM@HAD, and EMM@DJHAD were intravenously injected into the mice followed by observation at designated time points. As displayed in Figure S14B, free IR783 was rapidly eliminated and showed a very weak fluorescence signal at 24 h post‐injection, while NPs prolonged the circulation time of IR783 and enhanced the fluorescence intensity at tumor site. For IR783‐HAD and IR783‐DJHAD treated mice, the fluorescence of tumors synchronously increased and reached a similar maximum mean fluorescence intensity (MFI) at 6 h post‐injection (Figure 3A). Most notably, EMM@IR783‐DJHAD exhibited a superior tumoral IR783 signal compared to EMM@IR783‐HAD at 8 h post‐injection, as indicated in Figure 3B. In addition, the ex vivo biodistribution was investigated at 36 h post‐injection. The result in Figure S14C showed that all formulations displayed similar tissue biodistribution with a maximum accumulation of NPs in tumor tissue and maximum accumulation of IR783 in lungs and kidneys. The semi‐quantitative analysis results shown in Figure S14D were consistent with the fluorescence images. The above results suggested that EMM‐camouflage could drive nanodrugs to accumulate in pyroptotic tumor tissues, enhancing the tumor‐targeting effect, as illustrated in Figure S14A.

Based on excellent in vitro antitumor efficacy and superior targetability of EMM@DJHAD, we further evaluated in vivo tumor‐inhibiting effect of the nanoplatform. 4T1 bearing Balb/c mice were randomly divided into seven groups (*n* = 8 in each group from two batches) (Figure 3C). As shown in Figure 3D and Figure S15, EMM@JHAD and EMM@DHAD exhibited a partial tumor inhibition effect compared to the saline and EMM@HAD groups. The combination of DAC and JTC801 resulted in a more pronounced suppression of tumor growth. Among these combination therapy groups, EMM@DJHAD was more effective, with an average tumor volume of < 200 mm^3^. At the first endpoint on day 14, tumors from sacrificed mice (*n* = 5) were weighed and various parameters were characterized. Figure 3E showed that the average tumor weight displayed a similar tendency as the volume change. Moreover, hematoxylin and eosin (H&E) staining and terminal deoxynucleotidyl transferase dUTP nick‐end labeling (TUNEL) assay (Figure 3F) demonstrated that most serious malignant necrosis with cell fragmentation and nuclei shrinkage occurred in EMM@DJHAD group, although obvious cell apoptosis was also observed in DAC&JTC and DJHAD groups. In addition, the expression of Ki67, a cell proliferation marker, was significantly down‐regulated after EMM@DJHAD treatment (Figure 3F,G). Furthermore, cleaved caspase 3 and GSDME levels verified that pyroptosis was involved in EMM@DJHAD mediated superior antitumor efficacy (Figure 3H,I).

Remarkably, a drop in body weight was observed in the DAC&JTC treatment group (Figure S16). To explore the reason for the severe side effects, main tissue sections (heart, liver, spleen, lung, and kidney) were evaluated using H&E staining. Figure S17 displayed that no histological abnormality was found in heart with myocardial fibers, clear cell boundaries, and consistent cell shape. However, infiltration of inflammatory cells (blue arrows) could be observed in liver, spleen, and lung, suggesting that inflammatory changes were induced by DAC and JTC801. In addition, abnormalities in spleen sections with tubular epithelial cell denaturation, swelling, and cytoplasm rarefaction (yellow arrow) indicated impaired renal function. Other groups demonstrated invisible cardiotoxicity with neither noticeable body weight loss nor abnormality in main tissues (Figure S18). These results indicated that targeted delivery of chemotherapeutic agents was highly desirable for limiting the side effects in healthy tissues. Moreover, blood routine, hepatic, and renal function of mice treated with nanodrugs were used to evaluate biocompatibility. As displayed in Figure S19, the biochemical and hematological blood parameters were not significantly different statistically between EMM@DJHAD treated and healthy mice.

The antimetastatic activity of EMM@DJHAD was evaluated at the second endpoint on day 24 (*n* = 3). As shown in Figure 3J and Figure S20, a large amount of lung metastatic nodules was clearly observed in saline group, whereas only a few metastatic foci were observed in EMM@DJHAD treated group, suggesting efficient suppression of lung metastasis. The above findings confirmed that EMM@DJHAD nanoplatform with excellent biosafety exhibited outstanding therapeutic effects on tumor progression and metastasis.

### 2.4 Inhibition and analgesic efficiency in breast cancer bone metastasis model

**FIGURE 4:**
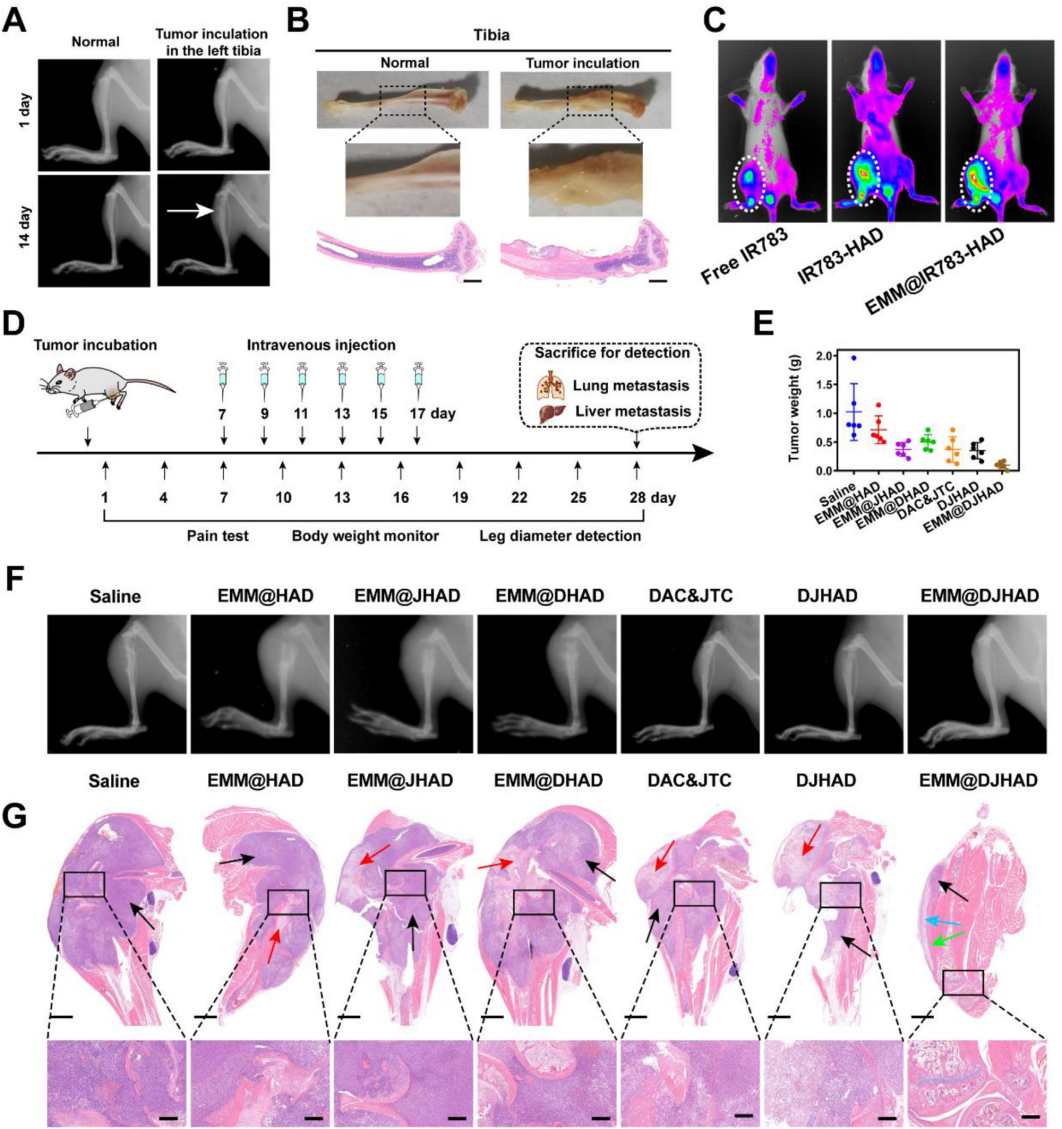
Anti‐tumor analysis on breast cancer bone metastases. (A) Radiographs of tibia bone on days 1 and 14 post‐injection. (B) Representative images and hematoxylin and eosin (H&E) staining of normal and tumor bearing tibia. (C) In vivo near‐infrared (NIR) images at 24 h. (D) Timeline of treatment schedule. (E) Tumor weight. (*n = 5*). (F) Radiographs of tibia bone. (G) H&E‐stained images of tumor‐bearing left hindlimb. Black arrows: tumor cells. Red arrows: apoptotic cells. Green arrows: fibroblasts and proliferation of capillaries. Blue arrows: proliferation of osteoblasts in the lateral cortex of peripheral bone. Scale bar on the top and bottom row indicated 2000 and 200 µm, respectively.

Intratibial injection of 4T1 cells was used to establish a breast cancer bone metastasis model. Radiographic imaging and histological analysis were used to track pathological changes in the hindlimbs.^[^ ^25^ ^]^ In Figure 4A, obvious osteolytic lesions were observed in the tumor‐bearing tibia compared to normal tibia on the 14th day post‐injection. Moreover, both anatomical changes and H&E staining of the tibia verified bone destruction with bone loss in cortex and medulla (Figure 4B), in line with tibia radiograph, confirming the successful establishment of the model.

Bone metastasis tropism of EMM camouflaged nanoplatform was examined in above mouse model using IR783 as a fluorescence tracer. Figure 4C indicated that the fluorescence intensity of bone metastatic lesions in EMM@IR783‐HAD group was the highest, although free IR783 and IR783‐HAD could also accumulate in tumor bearing tibia. As seen in Figure S21A, an obvious fluorescence signal was observed at tumor site after 1 h post‐injection in EMM@IR783‐HAD group, which increased over time and remained at a high level after 24 h. Meanwhile, free IR783 was rapidly eliminated and the fluorescence signal in tumor was much lower. IR783‐HAD accumulated slowly in the bone metastatic lesions and high distribution in lung and kidney was observed (Figure S21B). Quantitative MFI analysis at 24 h post‐injection further confirmed the strong bone metastasis‐targeting ability of EMM camouflaged nanoplatform, as shown in Figure S21C.

In view of bone metastasis targeting ability and promising antitumor efficacy of EMM@DJHAD, we evaluated its therapeutic effect on breast cancer bone metastasis, and experiment schedule is designed in Figure 4D. From Figure 4E, tumor tissues in EMM@DJHAD group were the lightest among all groups, which was consistent with the excised bone metastatic hindlimbs in Figure S22. Moreover, radiographic analysis prior to sacrifice demonstrated that EMM@DJHAD group exhibited smaller and fewer lesions than the other groups (Figure 4F). H&E staining demonstrated that tumor‐bearing legs in EMM@DJHAD group maintained their morphological appearance and structural integrity (Figure 4G). In contrast, enormous tumor space occupying lesions (black arrow) were observed in saline and EMM@HAD groups. However, different degrees of cell necrosis with nuclear shrinkage (red arrow) were observed in groups treated with different formulations. As a result, EMM@DJHAD showed superior antitumor efficiency, which was notably due to self‐cascade amplification targeting effect and synergism between DAC and JTC801.

Breast cancer is painless at its primary sites, but bone metastases cause excruciating pain, significantly reducing the life quality of patients. Here, we established two animal models to explore the effects of pain on tumor progression and metastasis (Figure S23A). One model was established via intra plantar injection of CFA into left hind paws of mice with ipsilateral tumor subcutaneously. For the second model, we mimicked neuropathic pain by spared nerve injury (SNI) with ipsilateral tumor subcutaneously.^[^ ^26^ ^]^ Pain behavior in mice was evaluated using a thermal nociception test (Hargreaves).^[^ ^27^ ^]^ Baseline of paw withdrawal latencies (PWLs) was measured before model construction.^[^ ^28^ ^]^ PWLs in the inflammatory and neuropathic pain groups were significantly lower than the basal PWLs, demonstrating the successful establishment of persistent pain models (Figure S23B). Compared to the pain‐free group, a significant increase in tumor volume and weight was observed in presence of persistent pain (Figure S23C,D). At the endpoint, a large number of nodules in lungs were observed in the groups with persistent pain (Figure S23F), which might account for their sustained weight loss (Figure S23E). Therefore, strategies where pain management synchronized with anti‐cancer therapy are urgent needed.

**FIGURE 5:**
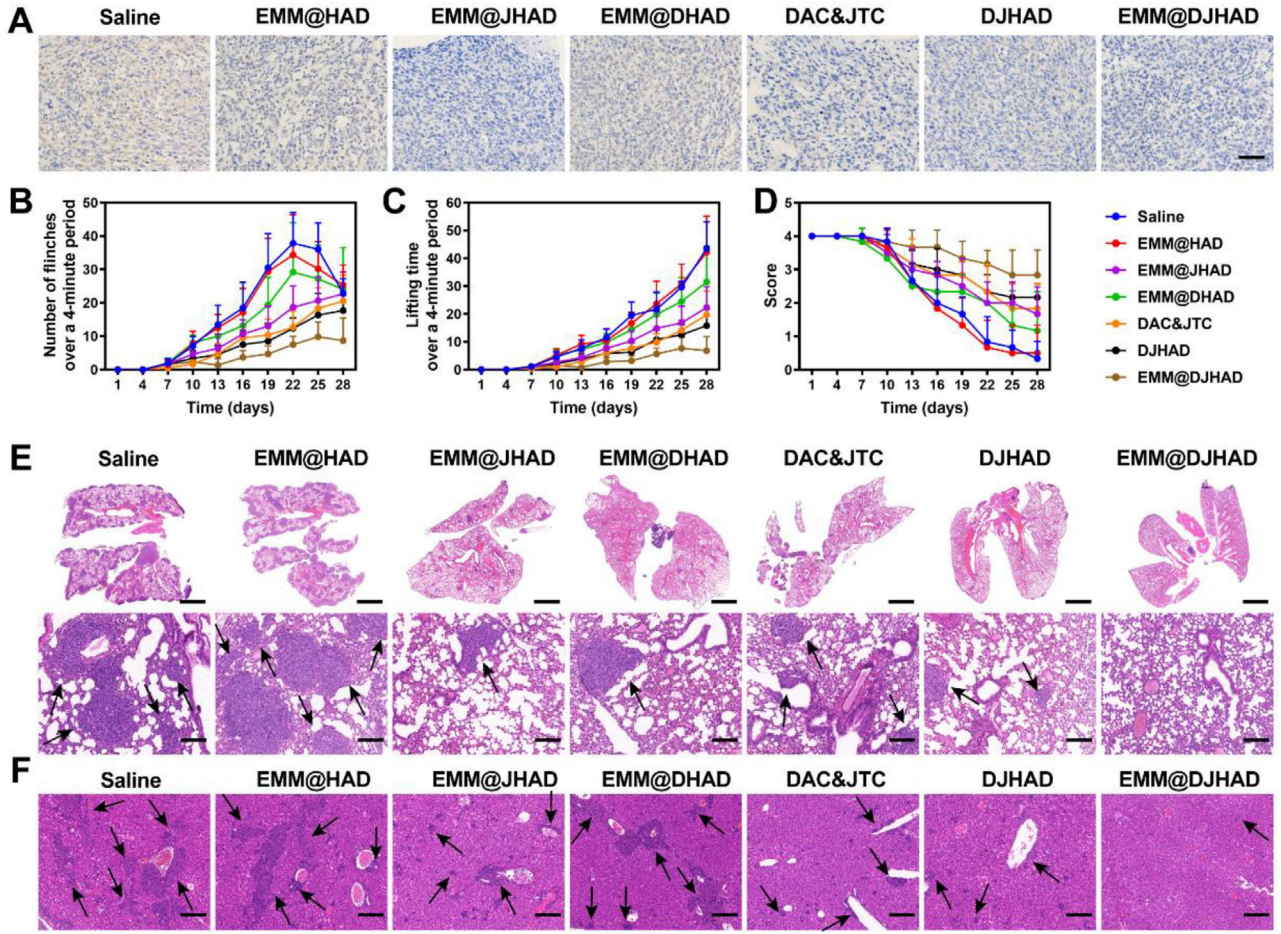
Analgesic effect anti‐metastasis of EMM@DJHAD (A) MOR expression, scale bar, 200 µm. (B) Spontaneous lifting time and (C) number of flinches of tumor‐bearing hind limb over a 4‐min observation period. (D) Score of 4‐min observation period. Limb use was scored: 4 = normal, 3 = limping, 2 = partial non‐use, 1 = substantial non‐use, 0 = non‐use. Representative hematoxylin and eosin (H&E) staining of (E) lung tissue sections and (F) lung tissue sections. Scale bar on the top and bottom row indicated 2000 and 100 µm, respectively. Data were presented as mean ± SD. Statistical analysis was conducted using one and two‐way analysis of variance (ANOVA). *p < 0.05, **p < 0.01, and ***p < 0.001. EMM, engineered macrophage membrane.

MOR is one of the most widely used targets expressed in central and peripheral nervous system. MOR has been proven to present and regulate several functions of tumor and stromal cells, including survival, proliferation, invasion, and angiogenesis.^[^ ^4^, ^9^ ^]^ In recent years, peripheral MOR analgesia under inflammatory conditions has attracted much attention, as it exerts significant analgesic effects with limited side effects. Nanoplatforms loaded with JTC801 could not only inhibit the opioid receptor µ−1 (OPRM1) expression in 4T1 tumor cells (Figure S24), but also suppress MOR expression in bone metastases (Figure 5A). The above results encouraged us to evaluate the analgesic effect of EMM@DJHAD based on the flinches number, spontaneous lifting time, and the movement scores of tumor‐bearing limbs over a 4‐min time period.^[^ ^29^ ^]^ Spontaneous lifting of the tumor‐bearing limb was first observed on day 9 post‐injection of 4T1 cells, and gradually increased over time, as shown in Figure 5B. A significant difference between EMM@DJHAD treatment groups and saline group was observed on day 13 (6 days post‐injection of the nanodrugs). Consistent with lifting time results, the number of flinches also gradually increased over time, reaching an average maximal level on 20th day (Figure 5C). Comparatively, EMM@JHAD, DAC&JTC, and DJHAD treatments partially attenuated the flinching compared to saline group, showing significant difference sometimes. Interestingly, the lifting time and number of flinches remained low in EMM@DJHAD treated group. The use of left hindlimb bearing tumor was scored to further evaluate pain level. Animals in saline group showed partial to substantial non‐use of the tumor‐bearing limb at the endpoint (Figure 5D). In comparison, EMM@DJHAD treated mice walked normally with occasional limping, suggesting the alleviation of cancer‐associated pain .

Confusingly, the body weight of the mice decreased sharply especially in saline and EMM@HAD groups (Figure S25). The underlying mechanism was investigated by anatomical observation of mouse visceral organ morphology and structure. A large number of nodules were observed in the lungs, which was significantly higher than that observed in the transplanted breast cancer model, as shown in Figure 5E and Figure S26. Similarly, liver metastasis was observed via pathological analysis (Figure 5F). Fortunately, both lung and liver metastases were attenuated by different formulations, among which EMM@DJHAD exhibited the most remarkable efficacy, with almost no nodules detected in the collected tissues. These results highlighted the potential of the EMM@DJHAD in analgesia and tumor inhibition, by suppressing bone tumors and attenuating secondary tumor metastasis.

## 3 CONCLUSION

In this current study, we constructed a biomimetic delivery system (EMM@DJHAD) for the concurrent management of breast cancer with bone metastasis and cancer associated pain. The nanoplatform could efficiently be home to the transplanted and bone metastatic breast cancer via inflammation‐directed chemotactic ability inherited from M1 macrophage. The released chemotherapeutic agents boosted chemotherapy by synergistically inducing tumor pyroptosis, thereby enhancing tumor targeting effect. EMM@DJHAD showed positive effect on tumor suppression and pain management, as demonstrated in the xenograft breast cancer and bone metastasis mouse model. Moreover, EMM biomimetic drug vector‐mediated precise delivery significantly reduced the toxicity of chemotherapeutic agents in normal tissues, demonstrating good biocompatibility. Determination of the involvement of other mechanisms in the antitumor and analgesic effects of EMM@DJHAD requires more in‐depth investigations in the further.

## 4 EXPERIMENTAL SECTION

Experimental details are provided in the Supporting Information.

## CONFLICT OF INTEREST STATEMENT

The authors declare no conflicts of interest.

## Supporting information

Supporting Information

## Data Availability

All the data associated with this study are presented in the paper or in the Supporting Information.

## References

[1] N. Harbeck , F. Penault‐Llorca , J. Cortes , M. Gnant , N. Houssami , P. Poortmans , K. Ruddy , J. Tsang , F. Cardoso , Nat. Rev. Dis. Primers 2019, 5, 66. 31548545 10.1038/s41572-019-0111-2

[2] a) Y. Gao , I. Bado , H. Wang , W. Zhang , J. M. Rosen , X. H. F. Zhang , Dev. Cell 2019, 49, 375; 31063756 10.1016/j.devcel.2019.04.012 PMC6506189

[3] a) R. L. Satcher , X. H. F. Zhang , Nat. Rev. Cancer 2021, 22, 85; 34611349 10.1038/s41568-021-00406-5 PMC10281546

[4] a) C. Yang , Y. Sun , X. Ouyang , J. Li , Z. Zhu , R. Yu , L. Wang , L. Jia , G. Ding , Y. Wang , F. Jiang , Pain Med. 2020, 21, 3443; 32914185 10.1093/pm/pnaa265

[5] K. P. Trayes , S. E. H. Cokenakes , Am. Fam. Physician 2021, 104, 171. 34383430

[6] a) F. A. Fisusi , E. O. Akala , Pharm. Nanotechnol. 2019, 7, 3; 30666921 10.2174/2211738507666190122111224 PMC6691849

[7] a) R. L. Wanderman , J. M. Hagedorn , Pain Med. 2021, 22, 523; 33155032 10.1093/pm/pnaa364

[8] B. Boettcher , B. Seeber , G. Leyendecker , L. Wildt , Fertil. Steril. 2017, 108, 207. 28669481 10.1016/j.fertnstert.2017.06.009

[9] a) Y. Li , G. Li , T. Tao , X. Kang , C. Liu , X. Zhang , C. Wang , C. Li , X. Guo , Cancer Lett. 2019, 453, 1; 30928385 10.1016/j.canlet.2019.03.038

[10] W. Liu , H. Liu , Z. Zhang , J. Huang , Dose‐Response 2019, 17, 1559325819882873. 31662712 10.1177/1559325819882873 PMC6794653

[11] a) Y. Chen , B. Li , X. Chen , M. Wu , Y. Ji , G. Tang , Y. Ping , Chin. Chem. Lett. 2020, 31, 1153;

[12] a) D. Jana , Y. Zhao , Exploration 2022, 2, 20210238; 37323881 10.1002/EXP.20210238 PMC10191001

[13] Y. Huang , Z. Guan , X. Dai , Y. Shen , Q. Wei , L. Ren , J. Jiang , Z. Xiao , Y. Jiang , D. Liu , Z. Huang , X. Xu , Y. Luo , C. Zhao , Nat. Commun. 2021, 12, 4310. 34262026 10.1038/s41467-021-24564-0 PMC8280231

[14] a) A. Schroeder , D. A. Heller , M. M. Winslow , J. E. Dahlman , G. W. Pratt , R. Langer , T. Jacks , D. G. Anderson , Nat. Rev. Cancer 2012, 12, 39; 10.1038/nrc3180 22193407

[15] a) J. Xue , Z. Zhao , L. Zhang , L. Xue , S. Shen , Y. Wen , Z. Wei , L. Wang , L. Kong , H. Sun , Q. Ping , R. Mo , C. Zhang , Nat. Nanotechnol. 2017, 12, 692; 28650441 10.1038/nnano.2017.54

[16] X. Zhou , N. Yan , E. J. Cornel , H. Cai , S. Xue , H. Xi , Z. Fan , S. He , J. Du , Biomaterials 2021, 269, 33172607. 10.1016/j.biomaterials.2020.120345 33172607

[17] Y. Zhang , K. Cai , C. Li , Q. Guo , Q. Chen , X. He , L. Liu , Y. Zhang , Y. Lu , X. Chen , T. Sun , Y. Huang , J. Cheng , C. Jiang , Nano Lett. 2018, 18, 1908. 29473753 10.1021/acs.nanolett.7b05263 PMC7470025

[18] C. N. Serhan , N. Chiang , T. E. Van Dyke , Nat. Rev. Immunol. 2008, 8, 349. 18437155 10.1038/nri2294 PMC2744593

[19] H. Zhao , L. Wu , G. Yan , Y. Chen , M. Zhou , Y. Wu , Y. Li , Signal Transduction Targeted Ther. 2021, 6, 263. 10.1038/s41392-021-00658-5 PMC8273155 34248142

[20] a) Q. Zhang , B. Zhu , Y. Li , Front Immunol. 2017, 8, 71; 28210259 10.3389/fimmu.2017.00071 PMC5288347

[21] a) P. Zhao , M. Wang , M. Chen , Z. Chen , X. Peng , F. Zhou , J. Song , J. Qu , Biomaterials 2020, 254, 120142; 32485591 10.1016/j.biomaterials.2020.120142

[22] a) Y. Liu , L. Li , J. Liu , M. Yang , H. Wang , X. Chu , J. Zhou , M. Huo , T. Yin , Biomaterials 2021, 267, 120481; 33189053 10.1016/j.biomaterials.2020.120481

[23] C. Gao , Q. Huang , C. Liu , C. H. T. Kwong , L. Yue , J. B. Wan , S. M. Y. Lee , R. Wang , Nat. Commun. 2020, 11, 2622. 32457361 10.1038/s41467-020-16439-7 PMC7251120

[24] a) Y. Xue , S. Dai , J. Liang , W. Ji , Mol. Pain 2020, 16, 174480692092924; 10.1177/1744806920929246 PMC7303503 32552357

[25] W. Meng , M.‐M. Hao , N. Yu , M.‐Y. Li , J. Q. Ding , B.‐H. Wang , H.‐L. Zhu , M. Xie , Mol. Pain 2019, 15, 174480691987181. 10.1177/1744806919871813 PMC6710711 31394961

[26] Y. S. Kim , M. Anderson , K. Park , Q. Zheng , A. Agarwal , C. Gong , Saijilafu, L. Young , S. He , P. C. LaVinka , F. Zhou , D. Bergles , M. Hanani , Y. Guan , D. C. Spray , X. Dong , Neuron 2016, 91, 1085. 27568517 10.1016/j.neuron.2016.07.044 PMC5017920

[27] M. Cheah , J. W. Fawcett , M. R. Andrews , Bio‐protoc. 2017, 7, e2506. 28920069 10.21769/BioProtoc.2506 PMC5600253

[28] J. Feng , S. Lepetre‐Mouelhi , A. Gautier , S. Mura , C. Cailleau , F. Coudore , M. Hamon , P. Couvreur , Sci. Adv. 2019, 5, eaau5148. 30788432 10.1126/sciadv.aau5148 PMC6374102

[29] F. Wang , L. Chen , R. Zhang , Z. Chen , L. Zhu , J. Controlled Release 2014, 196, 222. 10.1016/j.jconrel.2014.10.012 25456829

