# Supplementary material for: Dynamic regulation of drug biodistribution by turning tumors into decoys for biomimetic nanoplatform to enhance the chemotherapeutic efficacy of breast cancer with bone metastasis
Source: Exploration (Beijing). 2023 Jul 2;3(4):20220124. doi: 10.1002/EXP.20220124 (PMC10624374; doi:10.1002/EXP.20220124)
Supplement: Supplementary file 1 — Supporting Information [file EXP2-3-20220124-s001.docx]

**Dynamic Regulation of Drug Biodistribution by Turning Tumors into Decoys for** **Biomimetic Nanoplatform to Enhance the Chemotherapeutic Efficacy of Breast Cancer with Bone Metastasis**

Cuixia Zheng^1^, Dandan Zhang^1^, Yueyue Kong^1^, Mengya Niu^1^, Hongjuan Zhao^1^, Qingling Song^1^, Qianhua Feng^1,2,3^, Xingru Li^1^ and Lei Wang ^1,2,3*^

1. School of Pharmaceutical Sciences, Zhengzhou University, Zhengzhou 450001, P. R. China

2. Henan Key Laboratory of Targeting Therapy and Diagnosis for Critical Diseases, Zhengzhou, Henan Province 450001, P. R. China

3. Key Laboratory of Advanced Drug Preparation Technologies, Ministry of Education, Zhengzhou 450001, P. R. China

4. Translational Medical Center of Huaihe Hospital, Henan University, Kaifeng, China.

*Corresponding authors. Tel: +86 371 67781910 (Lei Wang)

E-mail addresses: wanglei1@zzu.edu.cn (Lei. Wang)

# Materials

Hyaluronic acid (molecular weights 10 kDa) was purchased from Bloomage Freda Biopharm Co., Ltd. Deoxycholic acid (DOCA) were obtained from Sigma-Aldrich (St. Louis, MO, USA). 1-ethyl-3(3-dimethylaminopropyl) carbodiimide (EDC), N-hydroxysuccinimide (NHS) and N, N'-Dicyclohexylcarbodiimide (DCC) were purchased from Aladdin Reagent Database Inc. (Shanghai, China). Decitabine (DAC) was obtained from Dalian Meilun Biotech Co., Ltd (Dalian, China). JTC801 was got from MedChemExpress (China). 4T1 breast tumor cells were bought from Shanghai Cell Bank, Chinese Academy of Sciences (CAS). Balb/c (female, 6-8 weeks) were provided by Zhengzhou Laboratory Animal Center, Chinese Academy of Sciences (CAS).

# Preparation of MM@DJHAD

## Synthesis of Hyaluronic Acid-Deoxycholic Acid (HA-DOCA) Conjugates

To synthesize DOCA-NH_2_, DOCA, DCC and NHS were dissolved in tetrahydrofuran at a mole ratio of 1:1.2:1.2. Under atmosphere of nitrogen, the mixture was stirred at room temperature and the precipitated dicyclohexylurea was removed via filtration after 12 h later. Succinimido DOCA was obtained by further precipitating the filtrate with n-hexane. The succinimido DOCA was dissolved in N, N-dimethylformamide, which was subsequent added dropwise into ethylenediamine. After 6 h reaction at room temperature, the precipitates were harvested after precipitated in distilled water, washed with distilled water and dried in a vacuum.

HA-DOCA conjugates were synthesized by chemical conjugation amino of DOCA-NH_2_ to the carboxylic acid of HA. To activate the carboxyl groups of HA, HA, EDC and NHS were dissolved in formamide with a mole ratio of 1:3:3. After stirring for 30 min at room temperature, DOCA-NH_2_ was added and reacted for another 24 h. The precipitate was obtained by precipitated in cold diethyl ether and dialyzed (MWCO 10,000 Da) for 48 h. The resulting product was freeze-dried and analyzed by proton nuclear magnetic resonance (^1^H NMR) spectroscopy (Bruker, Switzerland) and fourier transform infrared spectroscopy (FTIR) (Nicolet iS10, Thermo, USA)

## Preparation of DAC/JTC-HAD (DJHAD) nanodrugs

DAC and JTC801 co-loaded nanoparticles (DAC/JTC-HAD, DJHAD) were prepared by a dialysis method. HA-DOCA, DAC and JTC801 were disserved in formamide at a ratio 10:2:3. Then, the mixed solution was added dropwise to aqueous solution, and the solution appeared turbid in this process. The final solution was dialyzed (MWCO 8000~14000 Da) for 12 h to remove the unentrapped DAC and JTC801. The supernatant was filtered through a 0.45 μm microporous membrane. HAD, JHAD and DHAD NPs were prepared as the same protocol.

## Characterization of DJHAD

The hydrodynamic diameter and zeta potential of HAD and DJHAD were detected by dynamic light scattering (DLS) using Nano-ZS90 (Malvern Instruments, UK). The corresponding morphology was characterized by JEM-1230 transmission electron microscopy (TEM; JEOL, Japan). The entrapment efficiency (EE) was detected by high performance liquid chromatography (HPLC; Waters, USA) and calculated via formulas below: EE (%) = (weight of loaded drugs/feeding weight of drugs) × 100%.

## Preparation of EMM@DJHAD.

M1 macrophages were obtained by treating RAW264.7 with 100 ng/mL lipopolysaccharide (LPS; Sigma-Aldrich) and 40 ng/mL of interferon γ (IFN-γ; PeproTech). To obtain engineered M1 macrophage membrane (EMM), M1 macrophages were suspended in ice-cold TM buffer solution (pH 7.4; 10 mM Tris + 1 mM MgCl_2_) and subsequently sonicated 5 s for 4 times at a power of 100 W using an ultrasonication (JYD-650L, Xinzhi Inc., China) at 4°C. Then, 1M sucrose was mixed with cell homogenate to a final concentration of 0.25 M sucrose, and the mixture was centrifuged at 2000 × g and 4°C for around 10 min. The supernatant was collected after centrifugation at 3000 × g for 30 min at 4°C. Afterward, EMM was washed with ice-cold TM buffer containing 0.25 M sucrose and stored at − 80°C for further use.

Then, EMM@DJHAD was subsequently prepared by fusing EMM vesicles with DJHAD NPs via a mini-extruder for at least 20 times. The resulting solution was centrifuged at 3000 × g for 30 min to remove uncoated membrane, and was subsequently centrifuged at 12,000 × g for 5 min to remove soluble membrane proteins.

## Characterization of EMM@DJHAD

The hydrodynamic diameter, and zeta potential of EMM vesicles and EMM@DJHAD were detected by DLS. The corresponding morphology was characterized by TEM. The protein profiles in EMM and EMM@DJHAD were characterized by western blotting. The stability of EMM@DJHAD was evaluated by DLS.

The release profiles of DAC and JTC801 from EMM@DJHAD were studied at different pH in presence or absence of HAase (0.5 mg/mL), and detected by HPLC. The cumulative release rate of DAC and JTC801 was calculated using the following equation: Cumulative drug release (%) = (the amount of drug released at time/the initial amount of drug in the NPs) × 100%, the initial amount of drug in NPs.

## Inflammation targeting

Female Balb/c mice of 8~10 weeks old received a single intraplantar injection of complete freund's adjuvant (CAF) of 20 μL in left hind paw using an insulin needle to induce inflammation. Thermal nociceptive test was then performed on the ipsilateral inflamed hind paw.

The mice were *i.v.* injected with free IR783, MM@IR783-HAD and EMM@IR783-HAD, respectively. The fluorescent distribution was measured by an IVIS spectrum *in vivo* imaging system (IVIS, PerkinElmer, USA) at scheduled time points. Following last image acquisition, main tissues (heart, liver, spleen, lung, kidney, brain, and edema hind paw) were collected from sacrificed mice for *ex vivo* NIR imaging.

## Hemolysis assay

Fresh heparinized blood was collected from 8-week-old Balb/c mice. 2% red cell suspension was obtained when dilute erythrocyte with deionized water and gradient concentration of EMM@DJHAD. After incubation at 37℃ for 2 h, the samples were centrifuged and photographed. Erythrocytes diluted with deionized water were used as 100% hemolysis. The percentage of hemolysis was obtained according to the absorbance of samples at 540 nm and following formula: Hemolysis (%) =I/I_0_ × 100%. Where I was the absorbance in the presence of EMM@DJHAD, and I_0_ represented the absorbance in water.

# *In vitro* study

## Intracellular uptake and lysosomes escape study

4T1 cells were seeded into 12 well tissue culture plates (1× 10^5^ cells/well) and incubated overnight. Then FITC and EMM@FITC-HAD were added into each well according to the need of specific experiment. Afterward, the cells were collected to evaluate phagocytic uptake and lysosomal escape by flow cytometry and confocal laser scanning microscopy (CLSM) at scheduled time points.

## Evaluation of cytotoxicity

4T1 cells were treated with different concentrations of EMM@HAD, EMM@DHAD, EMM@JHAD and EMM@DJHAD. After treatment for 24 h or 48 h, cell viability was examined by CCK-8 assay using a microplate reader (Synergy H1, BioTek).

## *In vitro* Annexin V-FITC/PI assay

4T1 cells were incubated with EMM@HAD, EMM@DHAD, EMM@JHAD and EMM@DJHAD at concentration of DAC (2.5 μM) and JTC801 (5 μM) for 24 h. Then, cells were collected and stained with Annexin V-FITC/PI according to instruction (Beyotime Biotechnology). Flow cytometry was used to analyze the percentage of apoptosis cells.

## ATP and LDH release assay

4T1 cells were incubated with EMM@HAD, EMM@DHAD, EMM@JHAD and EMM@DJHAD at concentration of DAC (2.5 μM) and JTC801 (5 μM) for 24 h. The cell morphology was observed and photographed. The cell supernatant was collected to monitor released adenosine triphosphate (ATP) and lactic dehydrogenase (LDH). The amount of LDH was detected with a LDH Release Assay Kit (Beyotime Biotechnology) based on an INT chromogenic reaction catalyzed by diaphorase, and ATP with an ATP Assay Kit (Beyotime Biotechnology) based on luciferin-luciferase reaction.

## Western blotting

After treatment with different formulations, 4T1 cells were collected and lysed to extract proteins for further western blotting. The denatured protein samples were separated on an SDS-PAGE at equal amounts. After transferred to PVDF membrane and incubated with 5% skim milk, the membrane was cultured with primary and horseradish peroxidase (HRP)-conjugated secondary antibody. Finally, the protein band was observed by an UVP imaging system (Ultra violet Products Ltd., CA, USA).

The levels of apoptosis related protein (bax, bcl-2, cytoplasm c and cleaved caspase-3) and pyroptosis associated protein (GSDME-FL and GSDME-N) were detected by western blot assay as above.

## Calcein-AM/PI double stain

4T1 cells were treated with EMM@HAD, EMM@DHAD, EMM@JHAD and EMM@DJHAD at a concentration of DAC (2.5 μM) and JTC801 (5 μM). After incubation for 12 h, the cells were stained with calcein-AM/PI according to instruction (YEASEN Biotechnology). Then, the fluorescence was observed and recorded by a fluorescence microscopy (Olympus Flowview V1000, USA).

# Tumor inhibition efficacy in transplanted breast cancer

## *In vivo* biodistribution

To establish subcutaneous tumor model, 1×10^6^ 4T1 cells in 100 µL of saline were subcutaneously injected into Balb/c mice of 8~10 weeks old at right flank. When tumor size reached 300~500 mm^3^, the mice were *i.v.* injected with free IR783 IR783-HAD, IR783-DJHAD, EMM@IR783-HAD and EMM@IR783-DJHAD, respectively. The fluorescent distribution and intensity were measured by an IVIS spectrum *in vivo* imaging system at scheduled time points. Following last image acquisition, main tissues (heart, liver, spleen, lung, kidney, tumor) were collected from sacrificed mice for *ex vivo* NIR imaging.

## Antitumor efficacy in transplanted breast cancer.

When tumor size reached 80~120 mm^3^, mice were randomly divided into 7 groups (n = 8 in each group). The tumor-bearing mice were treated with saline, EMM@HAD, EMM@JHAD, EMM@DHAD, DAC&JTC, DJHAD and EMM@DJHAD by *i.v.* injection (DAC: 2.5 mg/kg, JTC801: 6.6 mg/kg), respectively. Treatments were performed every other day according to schematic illustration. The tumor volume and body weight of mice were continuous monitored for 14 days. The main tissues were collected on day 14 for further analysis and tumor weight was acquired after tumor issue isolated from sacrificed mice. At day 24, lung tissues were collected to detect lung metastasis by observing nodules.

# The inhibition efficacy and analgesic efficiency bone metastasis model

## Targeting effect of bone metastases

A mice model of bone metastasis from breast cancer was established by intra-tibia injection of 4T1 cells, as previously described. Briefly, female Balb/c mice of 8~10 weeks old were deeply anesthetized with pentobarbital sodium (45 mg/kg, intraperitoneal injection). Subsequently, tibia of left hind limb was carefully exposed after disinfection with 7% iodine and 75% (v/v) ethanol. And 10 μL of 4T1 cells (5 × 10^4^) were slowly injected into the intramedullary canal of bone using a 23-gauge needle. Syringe was left in the injection site for 3 additional minutes to prevent leakage of tumor cells. Then, injection site was sealed with bone wax and the wound was finally closed. Radiological and histological detections were used to evaluate the osteolysis after inoculation of 4T1 cells.

The mice were *i.v.* injected with free IR783, IR783-HAD and EMM@IR783-HAD, respectively. The fluorescent the distribution was measured by an IVIS at scheduled time points. Following last image acquisition, main organs (heart, liver, spleen, lung, kidney and tumor-bearing left hind limb) were collected from sacrificed mice for *ex vivo* NIR imaging.

## Anti-tumor analysis on breast cancer bone metastases

A mice model of bone metastasis from breast cancer was established by intra-tibia injection of 4T1 breast cancer cells, as above described. Tumor-bearing mice were randomly divided into seven groups (n = 6) intravenously receiving one of following samples via tail vein at 7th day post-injection: Saline, EMM@HAD, EMM@JHAD, EMM@DHAD, DAC&JTC, DJHAD, and EMM@DJHAD.

Pain associated behaviors were tested following tumor cells injection according to schematic illustration. Mice were placed individually in an open plexiglas squared chamber with diameter of 20 cm. After a 5-minute acclimation period, the spontaneous lifting time, the number of flinches and the movement scores were measured over a 4-minute observation period according to a previous report. Every lift of left hind limb not related to walking or grooming was considered to be one flinch, and the duration of lift was counted until the paw again touched walking surface. Limb use was scored: 4 = normal; 3 = limping; 2 = partial non-use of right hind limb; 1 = substantial non-use of right hind limb; 0 = non-use of right hind limb.

Body weight of tumor-bearing mice in each group were recorded throughout the experiments. Following last detection, radiographs of tibia bone were recorded. Then organs (liver, lung and tumor-bearing left hind limb) were collected from sacrificed mice for further examination.

# Persistent pain accelerates xenograft tumor growth of breast cancer in mice

Two preclinical animal models were established to explore the effects of pain on tumor. For the first model, mimicked inflammation pain by injection of CFA in plantar region with xenograft tumor subcutaneously. For the second model, we mimicked neuropathic pain by spared nerve injury with xenograft tumor subcutaneously.

Hargreaves test was used to assess hypersensitivity to thermal nociceptive stimuli. Experimental mice were placed individually in an open plexiglas squared chamber (diameter: 20 cm) on a transparent glass floor with A movable radiant heat source (Ugo Basile Plantar Test, Italy) positioned under the glass floor. Before testing, mice habituate in the chamber for 1 h. Then, radiant heat source was moved directly beneath plantar surface of the left hind paw, and time (in seconds) that elapsed from switching on radiant heat until paw withdrawal was measured automatically. A cutoff time of 20 s was established to prevent tissue damage.

The subcutaneous tumor volumes were recorded using following formula: (a × b^2^)/2, where a and b represent tumor length and width (in mm), respectively. The tumor weight was acquired after tumor issue isolated from sacrificed mice. The lung tissues were fixed in bouin’s fixative solution. The lung metastatic nodules were observed by dissecting microscope.

# Ethics statement

All the animal procedures were performed in compliance with the Regulations for the Administration of Affairs Concerning Experimental Animals of China, and all animal experiments were approved by the Animal Ethics Committee of Zhengzhou University (Permit No. SYXK (Yu) 2018-0004).

# Statistical Analysis

Data of all measurements including *in vitro* and *in vivo* presented as mean ± standard deviation (SD). Mean ± SD was calculated from independent experiments (*n* ≥ 3). Significance was indicated by *p* values < 0.05 (*), < 0.01 (**), < 0.001 (***), and P > 0.05 were regarded as significant.


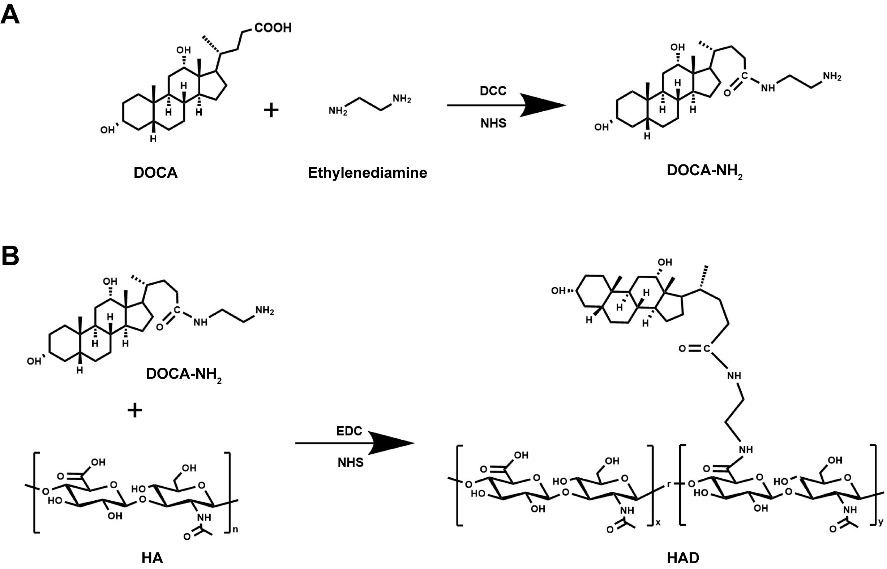


**Figure S1.** Synthetic procedure of HA-DOCA.


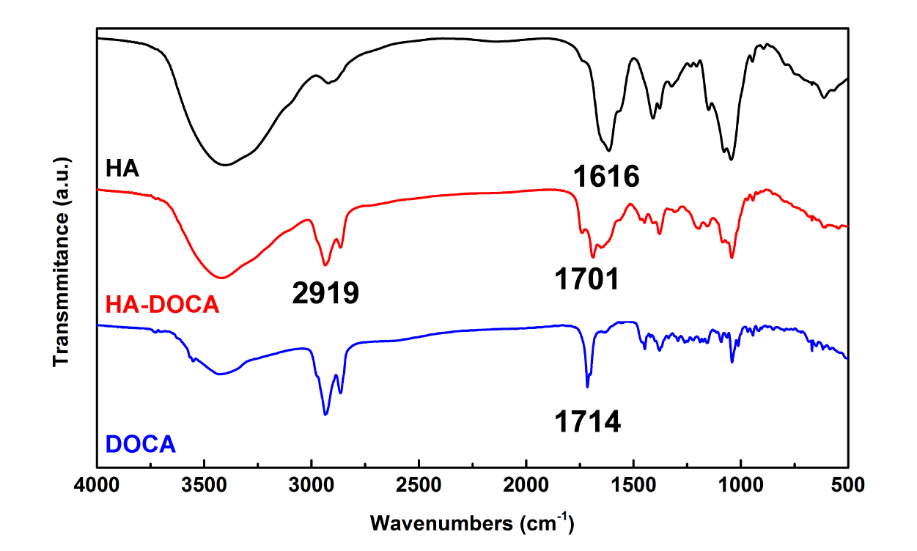


**Figure S2.** FTIR of HA, DOCA and HA-DOCA. In the analysis of FTIR, the increase of methylene absorption peak at 2919 cm^-1^ and an additionally peaks at 1701 cm^-1^ confirmed that HA-DOCA were successfully synthesized.


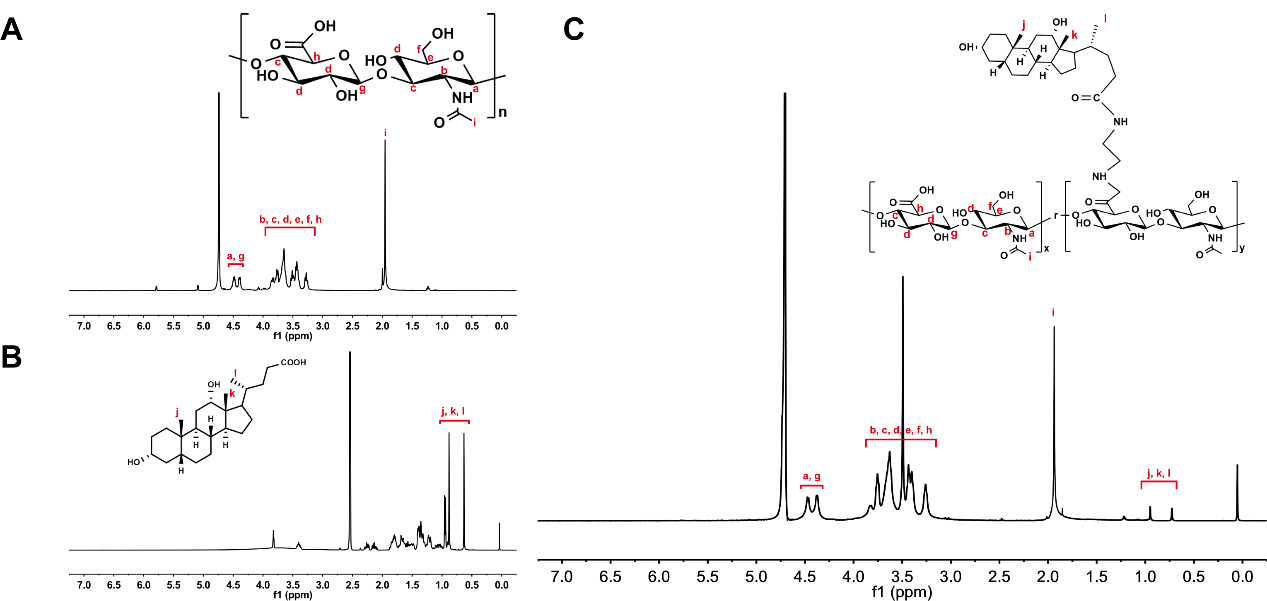


**Figure S3.** ^1^H NMR spectra of HA (A), DOCA (B) and HA-DOCA (C). In the analysis of ^1^H-NMR, d = 2.01 ppm and 3.28~4.75 ppm were specific peaks of HA. Successful introduction of DOCA into HA polymers was confirmed by the peaks of 0.67~1.60 ppm.


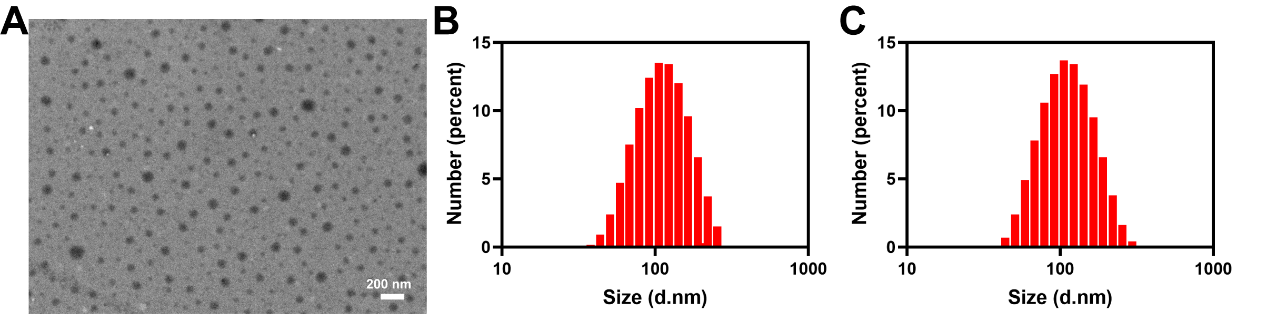


**Figure S4.** Characterization of HAD and DJHAD. (A) TEM image of HAD NPs. Size distribution of (B) HAD NPs and (C) DJHAD NPs.


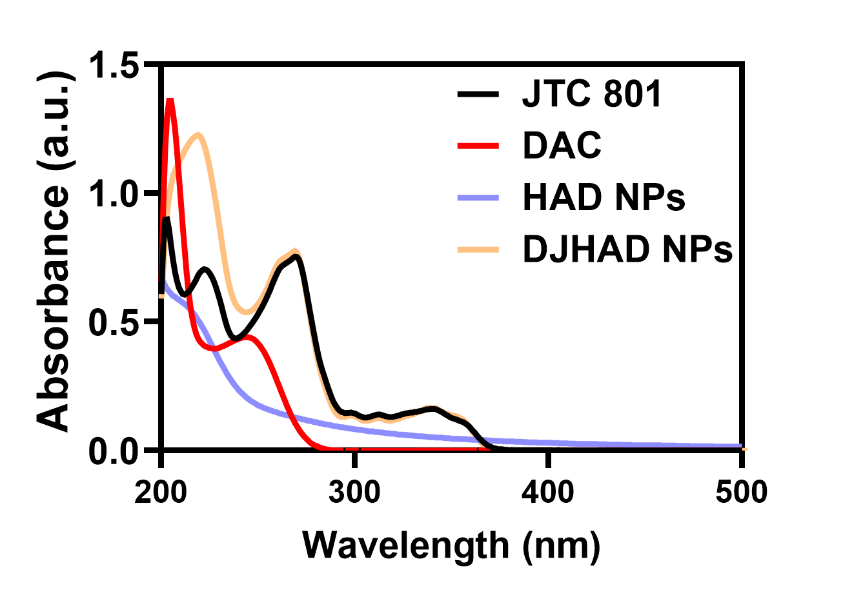


**Figure S5.** UV-vis-NIR absorption spectra


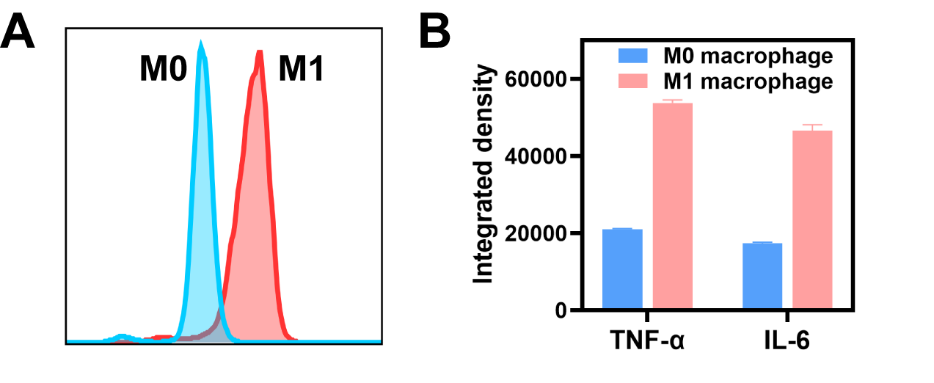


**Figure S6.** Macrophage polarization. (A) CD86 expression. (B) IL-12 and TNF-α in cell culture supernatant (*n=*3).


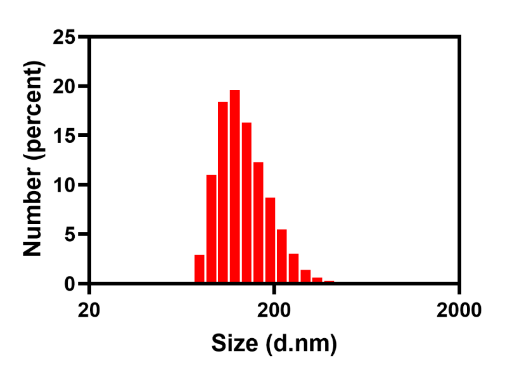


**Figure S7.** Size distribution of EMM@DJHAD.


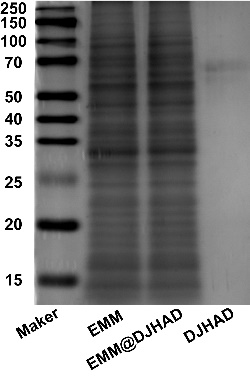


**Figure S8.** SDS-PAGE analysis of the proteins on EMM and EMM@DJHAD and DJHAD.


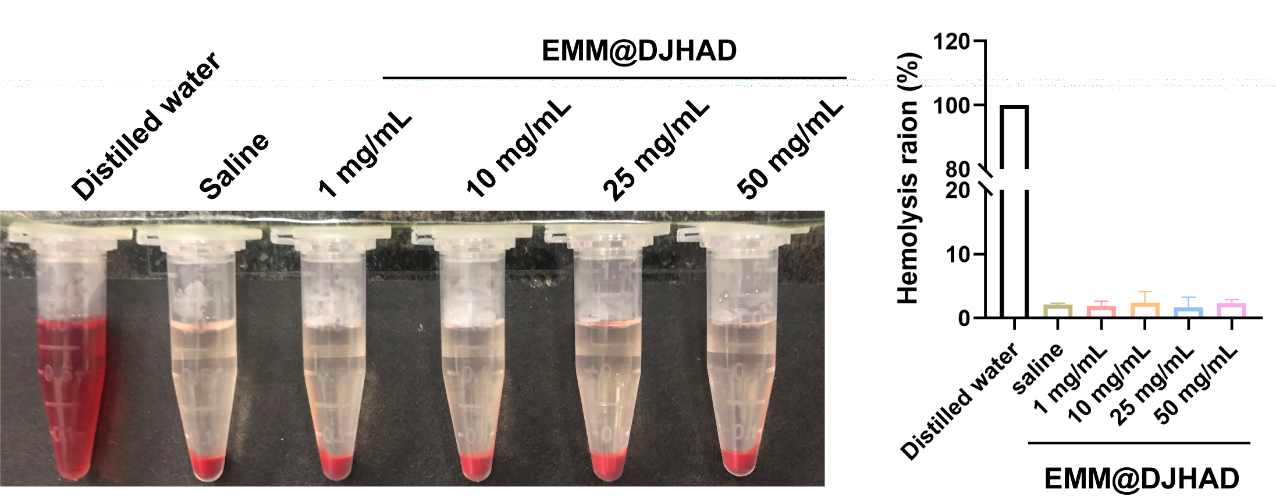


**Figure S9.** Hemolysis quantification at various concentrations of EMM@DJHAD.


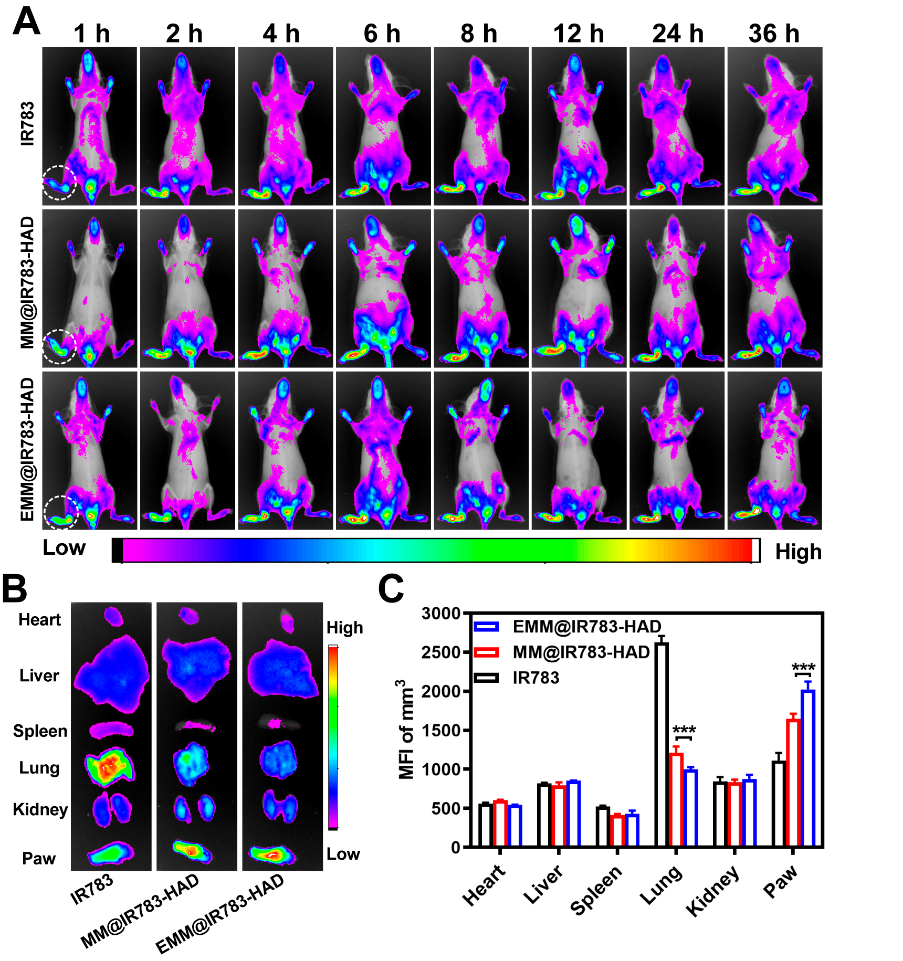


**Figure S10.** *In vivo* biodistribution in paw edema Balb/c mice. (A) *In vivo* near-infrared (NIR) fluorescence images. (B) *Ex vivo* fluorescence images and (C) IR783 fluorescence intensity of main tissues. ****p* < 0.001


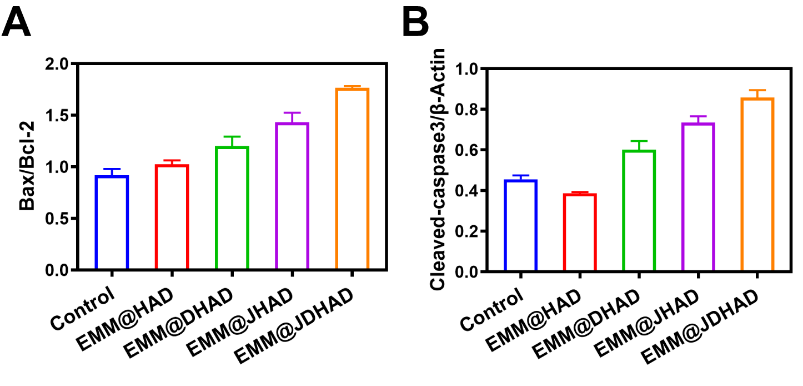


**Figure S11.** Quantitative analysis of (A) Bax/Bcl-2 and (B) Cleaved caspase-3


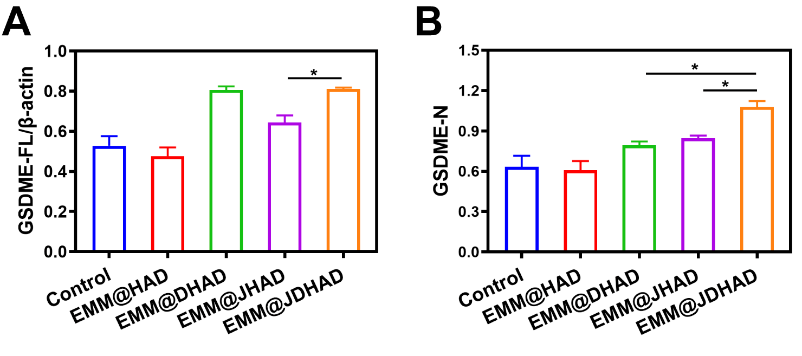


**Figure S12.** Quantitative analysis of (A) GSDME-FL and (B) GSDME-N. **p* < 0.05


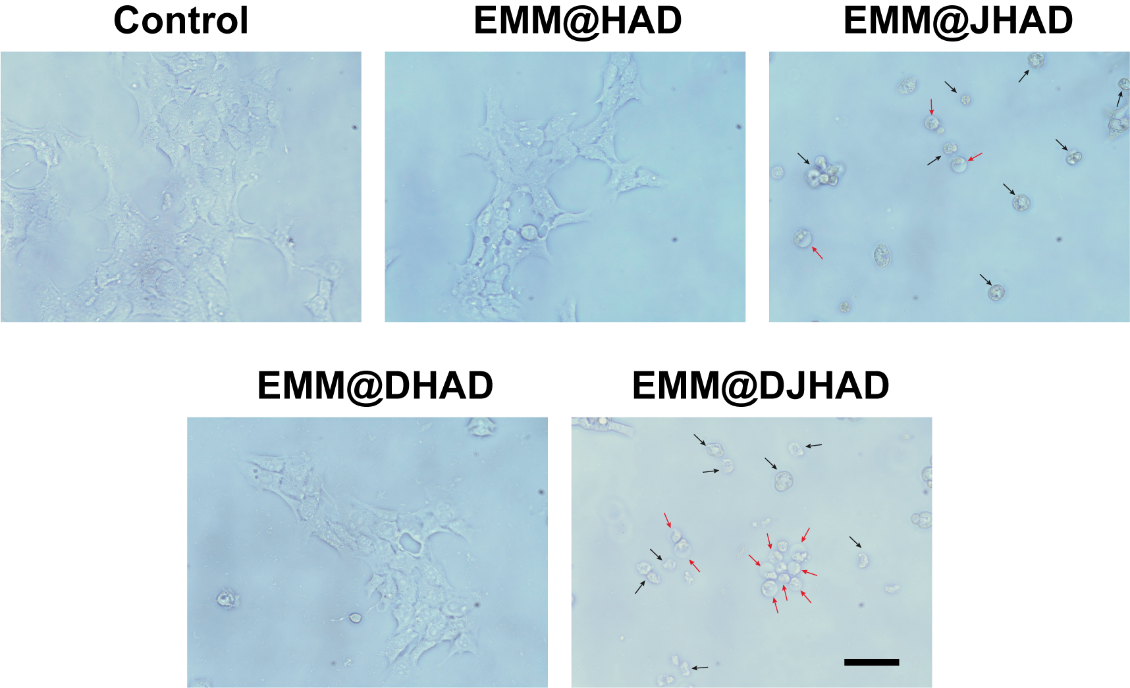


**Figure S13.** Representative high coverage images of 4T1 cells in bright-field microscopy, Scale bar: 100 μm. Red arrows, pyroptotic cells. Black arrows, apoptotic cells.


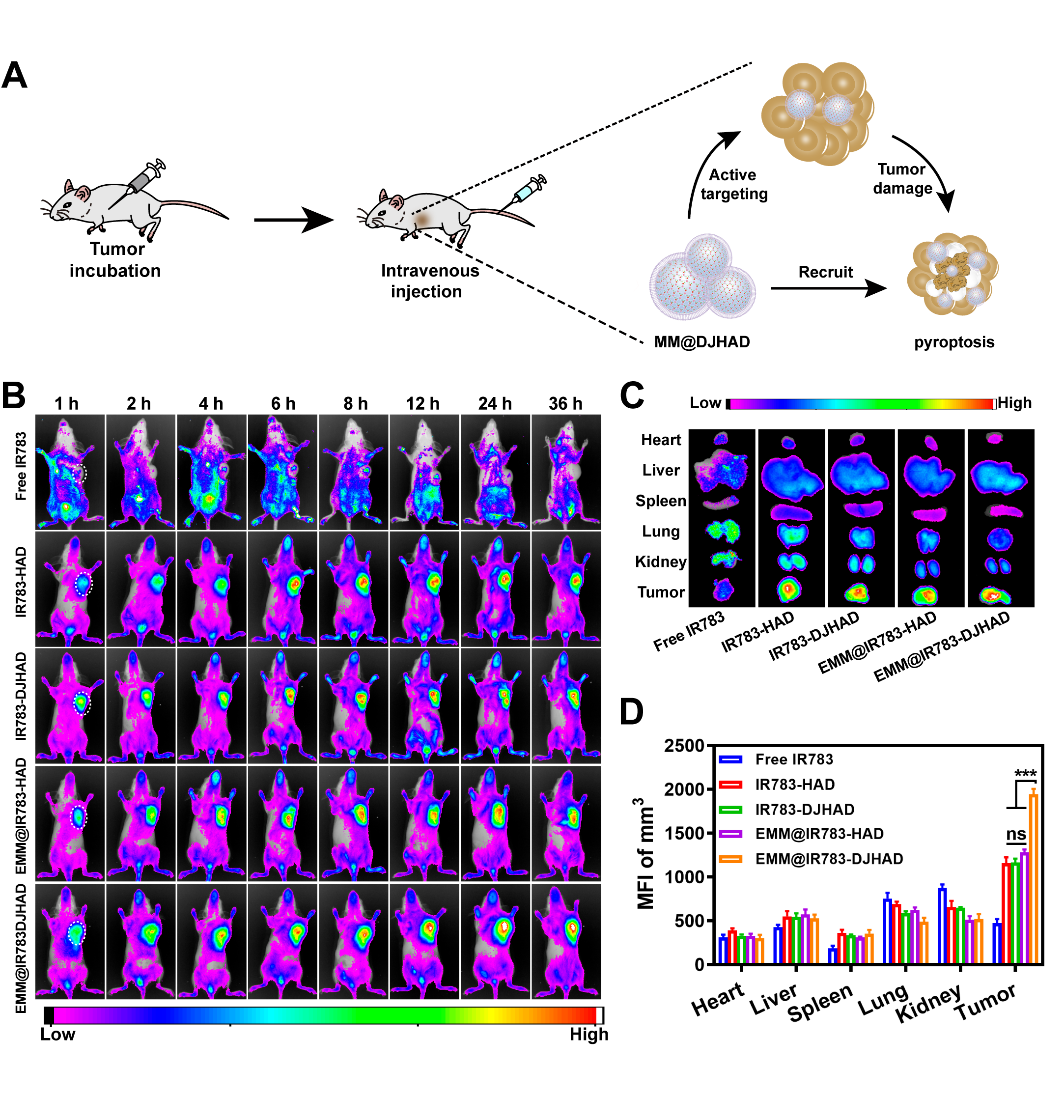


**Figure S14.** *In vivo* biodistribution in mice with transplanted breast cancer. (A) Mechanism of EMM-mediated targeting enhancement. (B) *In vivo* NIR images. (C) *Ex vivo* fluorescence images of main organs. (D) IR783 fluorescence intensity of main organs. Data represent means ± SD (*n*=3). ****p* < 0.001.


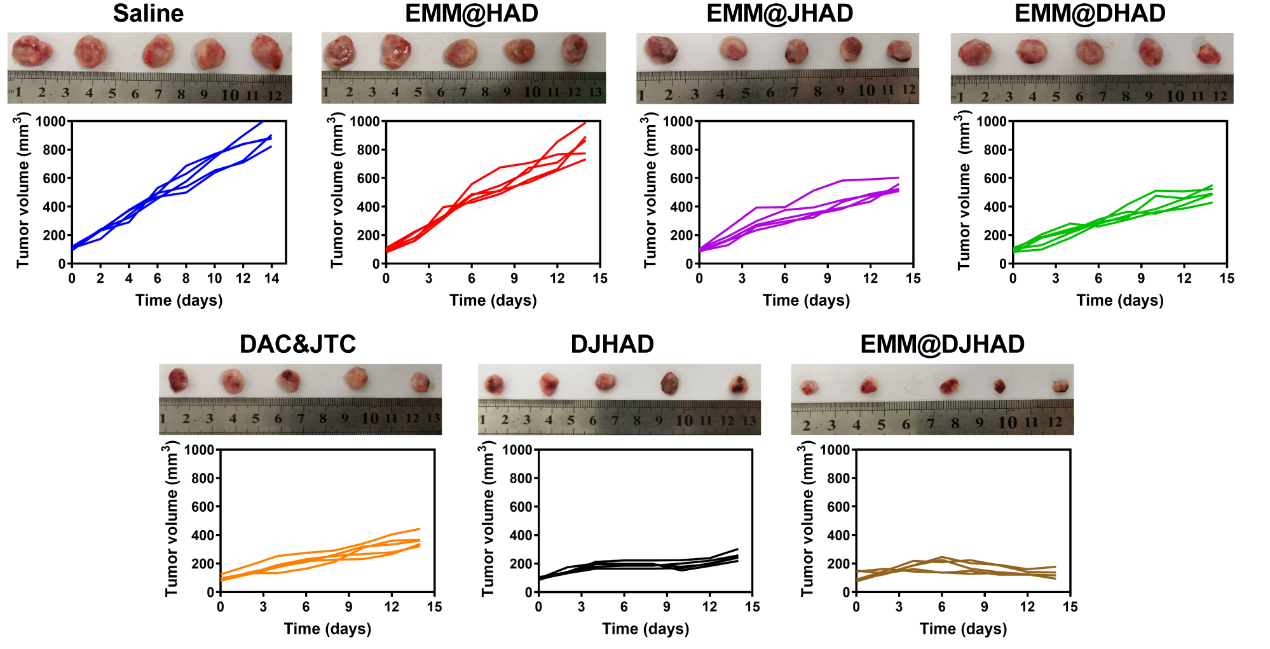


**Figure S15.** Individual tumor growth kinetics and photographic image of the excised tumors (*n*=5).


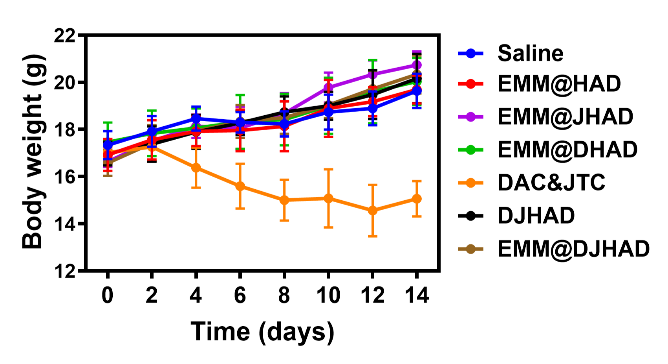


**Figure S16.** Dynamic body weight. (*n =* 5).


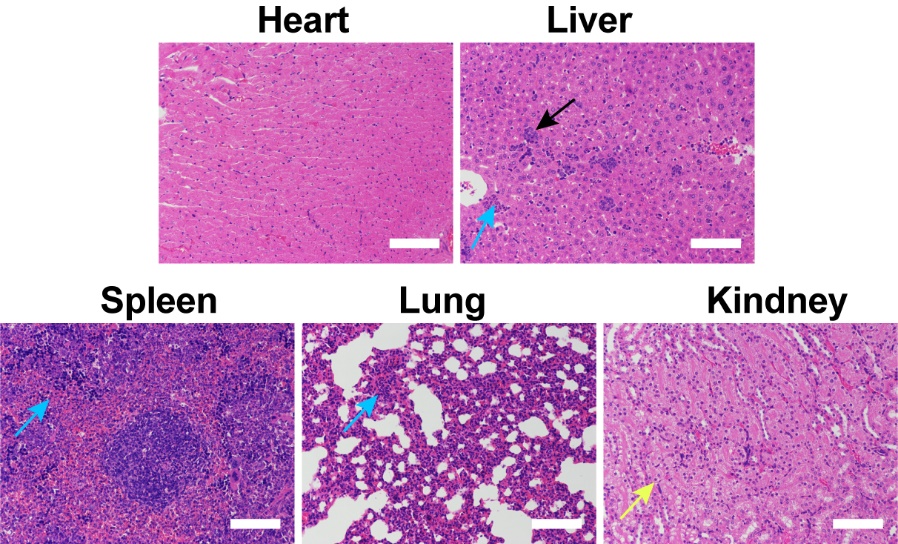


**Figure S17.** H&E staining micrographs of major organs collected from DAC&JTC group. Scale bar: 200 μm.


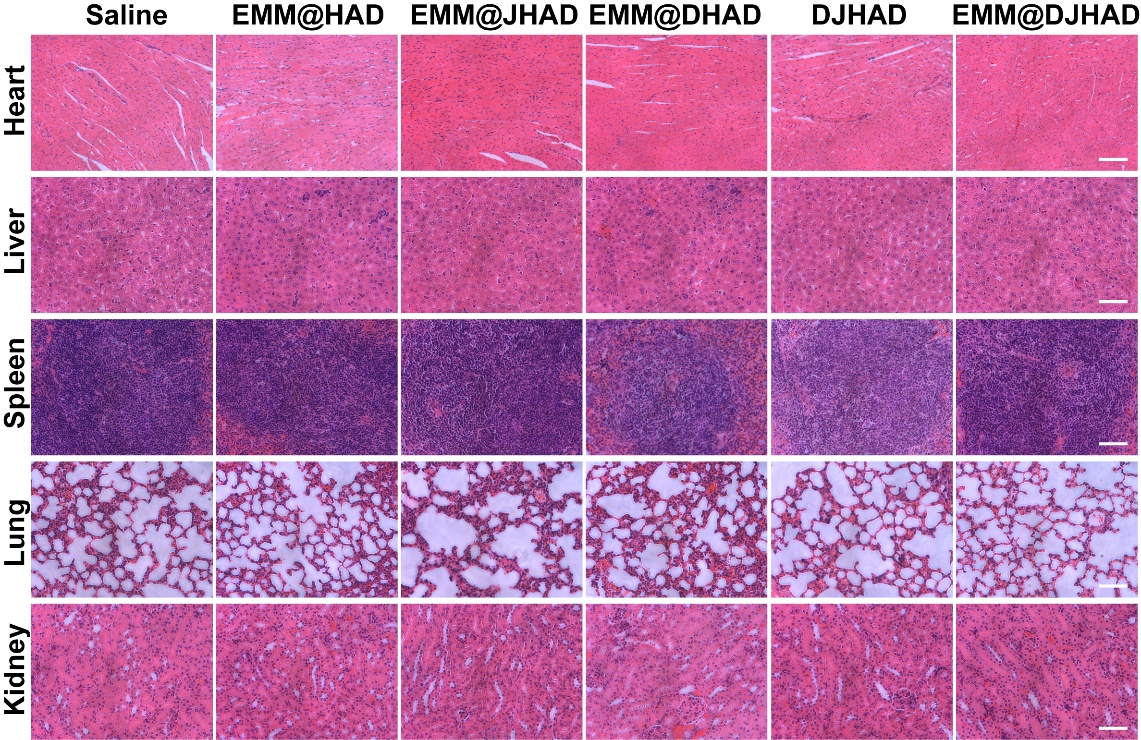


**Figure S18.** H&E staining micrographs of major organs after treatment with different groups. Scale bar: 200 μm.


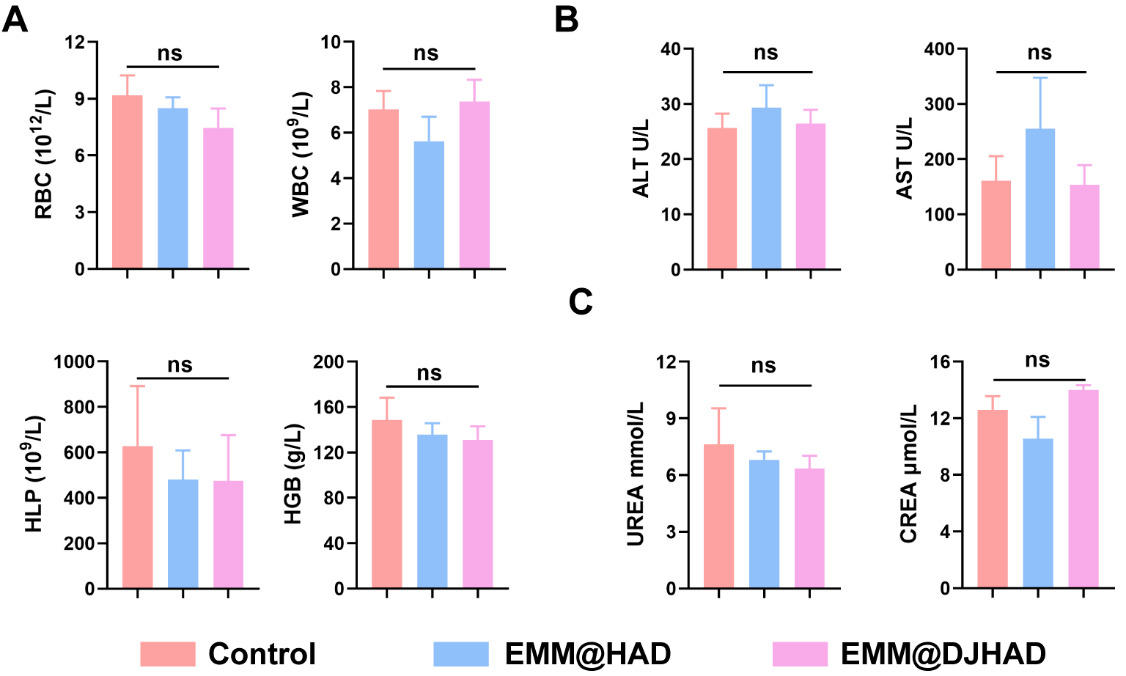


**Figure S19.** (A) The complete blood count studies. White blood cells (WBC), red blood cells (RBC), blood platelet (PLT). (B, C) Blood biochemistry analysis of serum. (B) Liver-function markers: alanine aminotransferase (ALT), aspartate aminotransferase (AST), (C) Kidney-function markers: UREA, CREA. The values are presented as the mean ± SD (*n* = 3).


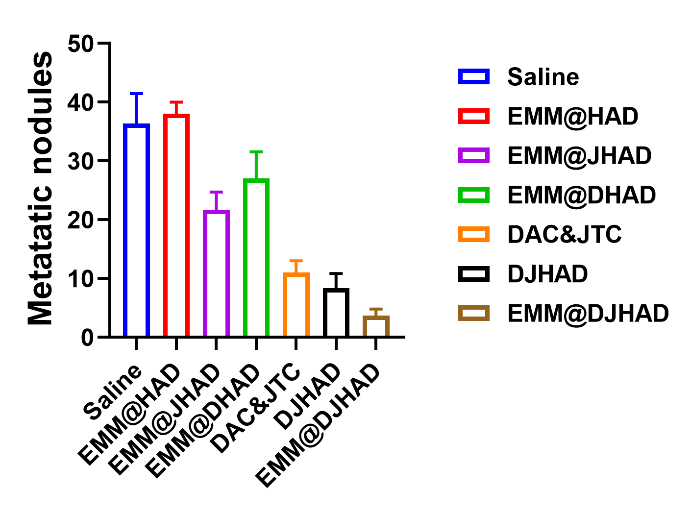


**Figure S20.** Quantification of pulmonary metastatic nodules in transplanted breast cancer.


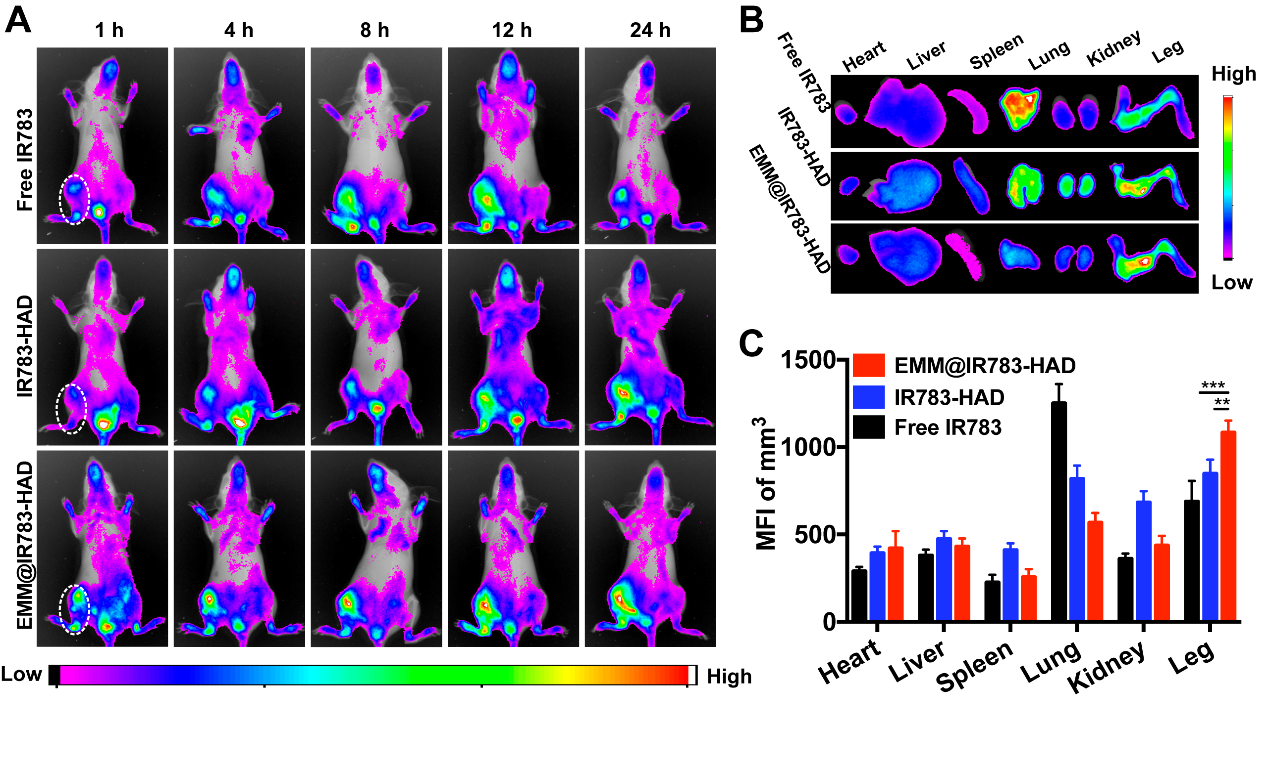


**Figure S21.** *In vivo* biodistribution in mice of breast cancer bone metastases. (A) *In vivo* near-infrared (NIR) fluorescence images. (B) *Ex vivo* fluorescence images and (C) IR783 fluorescence intensity of important tissues. Data was presented as mean±SD. (*n =*3) Statistical analysis was conducted using one-way ANOVA. ***p*<0.01, and ****p*<0.001.


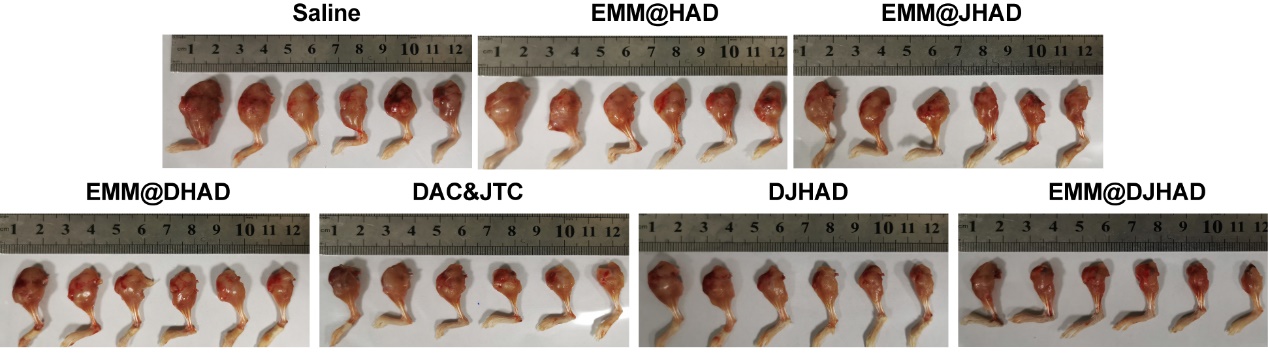


**Figure S22.** *Ex vivo* images of the representative bone metastatic hindlimbs (*n=*6).


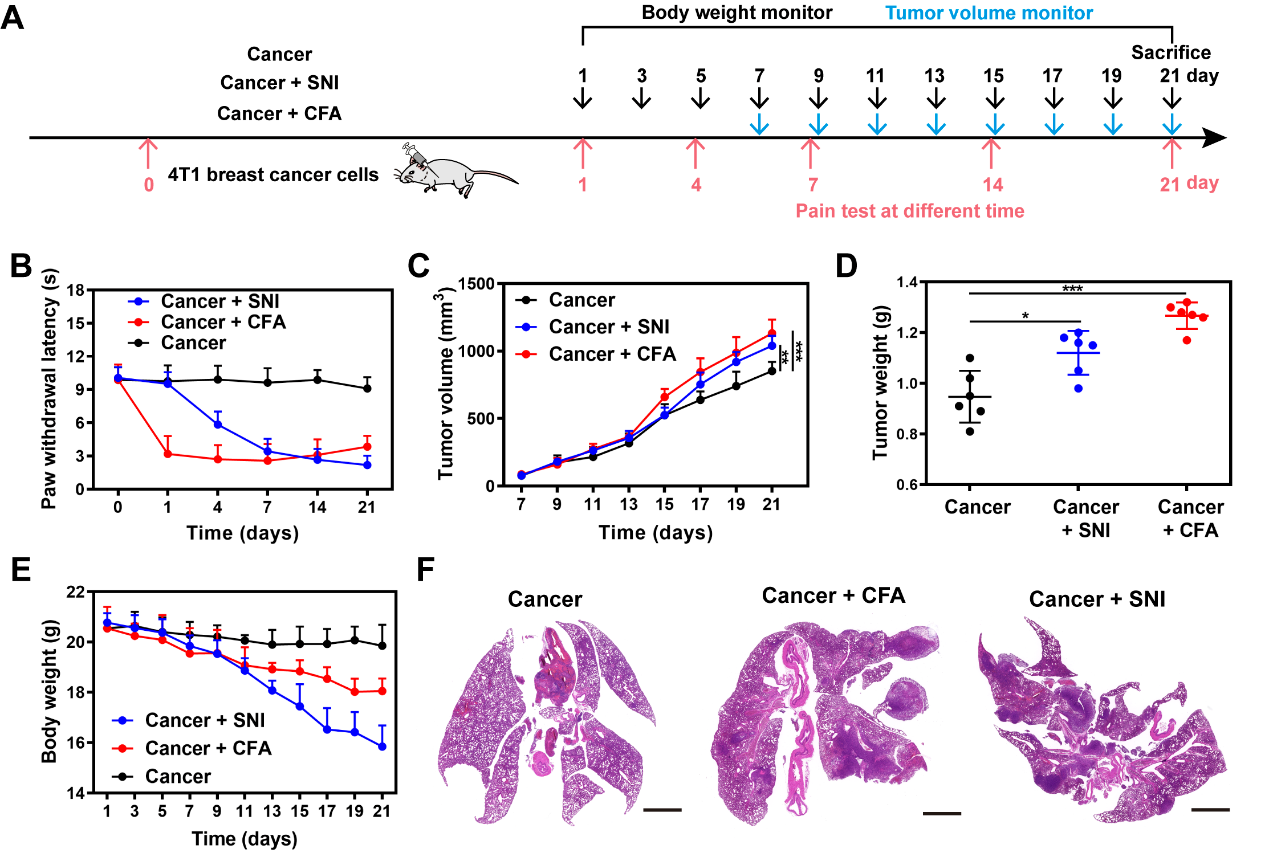


**Figure S23.** Persistent pain and tumor growth by ipsilateral inflammatory pain/persistent neuropathic pain model with subcutaneous breast cancer. (A) The time course of study design. (B) Hyperalgesic evaluated using Hargreaves test. (C) Time-dependent curves of tumor volume. (D) Tumor weight. (E) Dynamic body weight in different groups. (f) H&E staining of the whole lungs. Scale bar: 2000 µm. The values are presented as the mean ± SD (n = 6, **p* < 0.05, ***p* < 0.01, ****p* < 0.001).


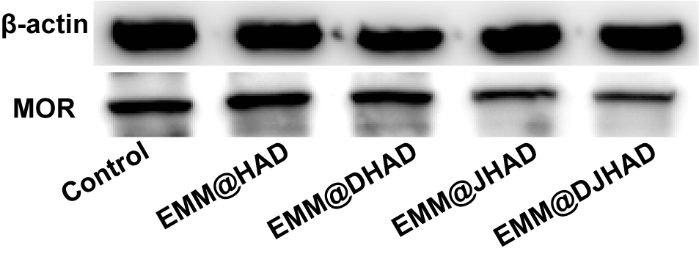


**Figure S24.** OPRM1 expression on 4T1 cells.


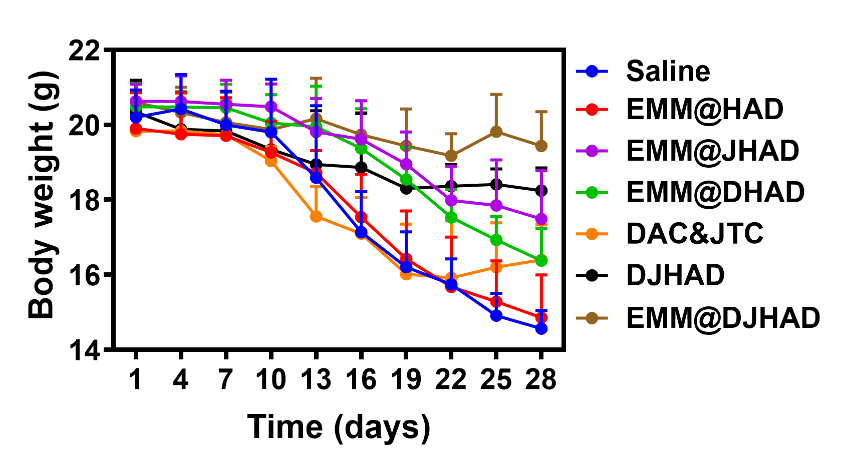


**Figure S25.** Body weight (*n=*6).


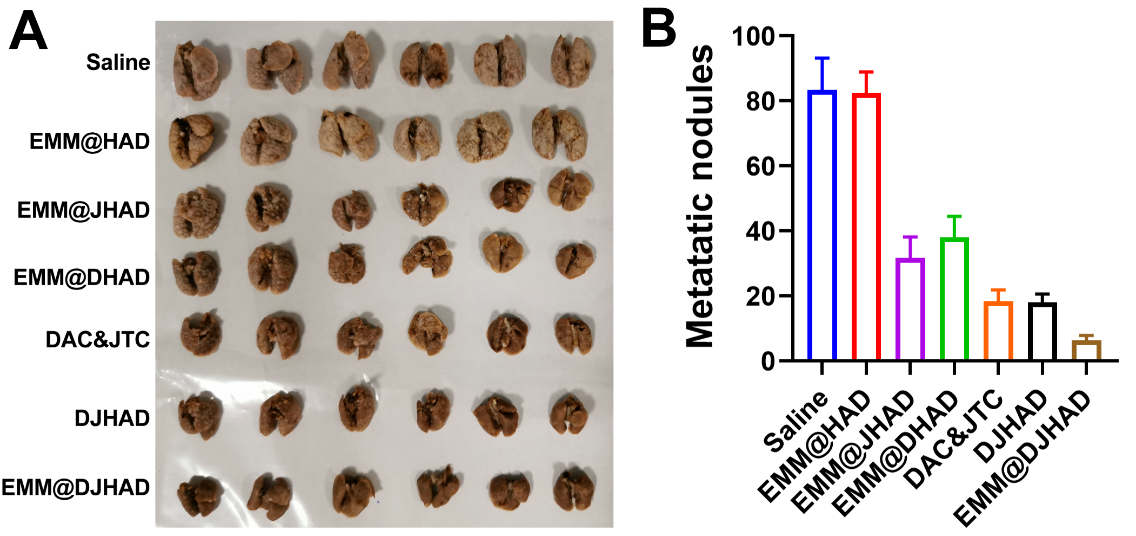


**Figure S26.** Lung metastases of different groups (*n=*6).
